# Novel Materials in Perovskite Solar Cells: Efficiency, Stability, and Future Perspectives

**DOI:** 10.3390/nano13111724

**Published:** 2023-05-24

**Authors:** Anup Bist, Bishweshwar Pant, Gunendra Prasad Ojha, Jiwan Acharya, Mira Park, Prem Singh Saud

**Affiliations:** 1 Department of Chemistry, Kailali Multiple Campus, Farwestern University, Mahendranagar 10400, Nepal; 2 Carbon Composite Energy Nanomaterials Research Center, Woosuk University, Wanju 55338, Republic of Korea; 3 Woosuk Institute of Smart Convergence Life Care (WSCLC), Woosuk University, Wanju 55338, Republic of Korea; 4 Department of Automotive Engineering, Woosuk University, Wanju 55338, Republic of Korea

**Keywords:** perovskite solar cell (PSC), efficiency, stability, TiO_2_ NPs

## Abstract

Solar energy is regarded as the finest clean and green energy generation method to replace fossil fuel-based energy and repair environmental harm. The more expensive manufacturing processes and procedures required to extract the silicon utilized in silicon solar cells may limit their production and general use. To overcome the barriers of silicon, a new energy-harvesting solar cell called perovskite has been gaining widespread attention around the world. The perovskites are scalable, flexible, cost-efficient, environmentally benign, and easy to fabricate. Through this review, readers may obtain an idea about the different generations of solar cells and their comparative advantages and disadvantages, working mechanisms, energy alignment of the various materials, and stability achieved by applying variable temperature, passivation, and deposition methods. Furthermore, it also provides information on novel materials such as carbonaceous, polymeric, and nanomaterials that have been employed in perovskite solar in terms of the different ratios of doping and composite and their optical, electrical, plasmonic, morphological, and crystallinity properties in terms of comparative solar parameters. In addition, information on current trends and future commercialization possibilities of perovskite solar have been briefly discussed based on reported data by other researchers.

## 1. Introduction

### 1.1. Perovskite Solar Cells

Energy demand is rising dramatically every day as the human population increases. As a result, the most widely used energy sources, such as coal, oil, diesel, and petroleum, are on the verge of becoming extinct. On the other hand, the emission of pollutants from the combustion of fossil fuels is the major cause of global warming. Therefore, it is eagerly required to promote renewable energy. The sources of renewable energy, such as solar, wind, tidal, geothermal, biomass, and hydrological energy, could replace fossil fuels to meet the world’s energy needs. Among them, solar energy is the most important due to its resource-free and zero-pollution. Since the invention of solar energy, silicon-based solar cells have been the only ones to be commercially successful. Unfortunately, it suffers from high costs, complex fabrication processes, and toxic byproducts. Therefore, other solar technologies, including cadmium telluride (CdTe), copper-Indium gallium diselenide (CIGS), dye-sensitized solar cells (DSSC), perovskite (PSC), and organic solar cells, are currently being researched and developed [1]. Among them, using perovskite semiconductor materials in solar systems has gained considerable focus to tackle the issues associated with the use of silicon materials in solar cells. Russian mineralogist L.A. Perovski developed the idea of perovskite [1,2]. This idea has defined the ABX_3_ crystal’s latter structure, where X represents oxygen or a halogen (anions), while A and B are cations. In this lattice structure, the twelve X anions that fill the cube’s octahedral site are shared by a massive A cation, while the little B cation molecule is shared by six X anions, which stabilizes the octahedral crystal structure. Among several perovskites, the oxides have attracted much attention due to their ferroelectric or superconductive features [2]. Due to its layered structure, the other type of perovskite, known as halide perovskite, has also gained favor since it boosts dimensionality.

Organic-inorganic perovskite solar cells have drawn greater interest in recent years because they have a higher power conversion efficiency (PCE) than that of a few first-generation (wafer-based), second-generation (thin-film-based) and third-generation (organic/inorganic) solar technologies [3]. In the laboratory-prepared perovskite solar cells, the PCE is higher than that of polycrystalline silicon and amorphous silicon-based first-generation solar cells. Similarly, perovskite has a higher efficiency than second-generation solar, such as amorphous and microcrystalline, and third-generation solar such as dye-sensitized and organic solar. As in the literature, the PSC’s current highest efficiency rate is 23.7% [3] and 25.2% [4]. The ease of manufacture, cost-effectiveness, time and material savings, and widespread use of the product are further reasons to pay attention. The high absorption coefficient, prolonged charge carrier diffusion interval, high charge carrier mobility, and flexible band gap are the main benefits of organic-inorganic perovskite solar cells [5]. Researchers’ attention may be drawn to these hybrid PSC structures because of their improved morphology and fruitful interactions between the light-absorbing layers (perovskite) and the electron transporting layer (ETL), the hole transporting layer (HTL), the counter electrode (CE), and the substrate/ETL [6]. Moreover, the ability to process at low temperatures and the incorporation of flexible and scalable materials or substrates are innovative features of PSCs. The PSCs adapt simple and affordable deposition methods such as slot-die coating, ink printing, doctor blade, spin coating, and screen printing [7]. One of the best and most novel perovskite layer deposition methods is called electrodeposition, which provides a uniform coating of the active layer with the help of an electric current and contains a salt solution. This deposition for the perovskite layer consists of three steps: (i) electrodeposition of PbO_2_, (ii) transformation of PbI_2_, and (iii) transformation of PbI_2_ into MAPbI_3_. The chlorinated electrodeposition is considered the best approach for PSC due to the chlorine’s incorporation into the active layer. Chlorination improves morphology, charge carrier distribution length, crystallinity, grain size, stability, and efficiency [8]. In PSCs, the primary goal is to reduce the cost of the device and improve its environmental impact by modifying it to use naturally existing carbon, such as carbon black, graphite, shot, and carbon prepared from various plants. Certain nanomaterials, including TiO_2_, ZnO, SnO_2_, doping, and composites, which might be used in ETL, HTL, and CEs, can offer scalability and flexibility. The nanomaterials have been applied in the diverse fields of optics and electronics. In perovskite solar cells, a material such as plasmonic NPs may improve solar parameters by inducing optical and electrical properties. The plasmonic NPs can be incorporated into the perovskite as a border-adapting substance. Plasmonic NPs used in the light-absorbing layer or charge transporter of perovskite solar improve optical and electrical properties, stimulate charge carrier transport, and promote charge collection ability [9]. In the interior structure of perovskite, plasmonic NPs, such as AuNPs, can exhibit exceptional thermal and chemical stability. In the visible-to-near-infrared (Vis-NIR) region, it maintains the localized surface plasmon resonance. Its optical (near- and far-NIR) effect can offer light scattering and quick absorption, and enhance the existence of an electromagnetic field on the AuNPs’ surface. Moreover, the size, shape, and dielectric environment of this AuNP surface have a significant impact on the electromagnetic effect. In addition, plasmonic Au NPs incorporated either in the light-absorbing layer or transporter layer provide a better route to focus incident photons in the light-absorbing layer through localized surface plasmon resonance (LSPR). Incorporation of these sorts of materials in PSC may be beneficial for future research and development [10]. In order to increase the PSC’s ability to absorb light, a number of materials (PEDOT:PSS, carbon nanotubes, nanowires, graphene, fullerene, quantum dots, composites, and doping) have been inserted in the different layers of the PSC [10]. Another sort of material is called a luminescent material, which has good light absorption across the solar spectrum. These materials absorb high energy and convert it into photons with low energy at a particular wavelength. For instance, sodium europium fluorine (NaEuF_4_) NPs are luminescent materials that absorb high-energy photons and convert them into one or more low-energy photons. It means NaEuF_4_ NPs used in PSC absorb ultraviolet (UV) light and transform it into visible light [11]. The NaEuF_4_ NPs were incorporated into the TiO_2_ mesoporous layer of PSC. The solar with NaEuF_4_ and without NaEuF_4_ were compared in terms of efficiency, and NaEuF_4_ was found to be more efficient. The best efficacy for the cell incorporating NaEuF_4_ is 14.51% [11]. Due to the abundant availability of some unique carbonaceous materials, the formerly employed HTL and ETL have been supplanted by carbon nanotubes (CNTs) [12], graphene [13], shoot-based carbon, carbon black [14], and graphdiyne [15]. These materials mostly replace the conventional HTL and CE materials (CuSCN, CuS, NiO, etc.) and (Au, Al, and Ag), respectively. They produce HTL-free perovskite by adding the aforementioned components since HTLs are expansive and restrict electrical flow to nearby electrodes. However, because of their transparency, mechanical flexibility, stability, hydrophobic nature, cost-effectiveness, and ease of production, these carbon nanostructures outperform the limitations of traditional materials. These materials can have a superior potential for producing stable and rigid PSCs [7]. The carbonaceous materials used in CE cannot compete with Au and Ag-based CE in terms of efficiency. The conductivity of these materials is extremely high compared to carbon and CE. However, widespread application of these materials is limited in PSCs due to the following reasons: (i) high vacuum process; (ii) long-term thermal process in a vacuum; (iii) single use of these materials leads to product wastage; (iv) environmental pollution; and (v) limited resources of these materials increase the cost of PSC [16,17]. To reduce the cost of pure novel metal-based CE, a composite of Ni/Au has been employed in PSC, which achieved 13.88% efficiency [18]. The deposition of Au as CE requires high thermal processing that is complex, laborious, and costly. To overcome the problems associated with Au CE, the nanopropus Au-CE has been tried as a superior, viable, and efficient alternative [17]. Recently, cathode interlayer (CIL) has been immersed as a novel approach in ETL and CE. This is made up of polymers, small organic molecules, metal oxides, salts of alkali, and two-dimensional materials. These materials are widely used for hole collection purposes in the form of high-work-function materials as anodes. However, low-work-function material is used for collecting electrons at the cathode of the inverted PSC. These have an organometallic framework whose electrical properties can be conducted by metals in the center and ligands surrounding the metals. The core of metal and chelating ligands potentially regulates the interface of PSC. However, the best efficiency for PSC containing organometallic complexes such as CIL with Ag as the central metal for inverted PSC is 21.2% [19]. This technique may be one of the better alternatives to pristine, novel metal-based CE [19].

### 1.2. Working Mechanism of Perovskite Solar Cells

**Figure 1:**
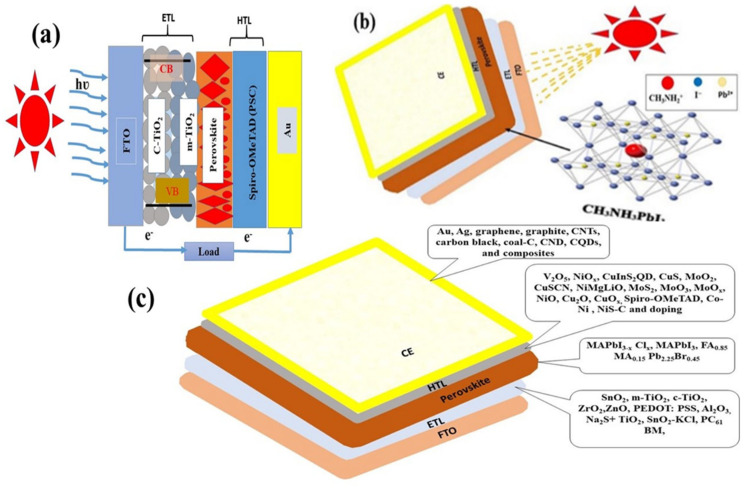
Pictorial representation of Perovskite solar cell. (**a**) Different constituents of solar connected through load, (**b**) typical position of perovskite with crystal structure, and (**c**) possible modification of different components of PSC.

Every layer in the perovskite solar cell, from top to bottom, creates a sandwich-like appearance. The substrate (front electrode), the electron transport layer (ETL), the light absorption layer (perovskite layer or active layer), the hole transport layer (HTL), and the counter electrode (CE) or back electrode are the five layers that make up the whole cell. Perovskite is a highly active layer that absorbs the incident photons and then separates electrons and holes. The most commonly used materials for the ETL layer are c-TiO_2_, m-TiO_2_, SnO_2_, ZnO, or other similar materials [20,21,22]. The working of PSC, its constitutes, and possible modified materials have been illustrated in Figure 1. 

The electrons diffuse in the direction of the substrate (FTO, TCO, or ITO) with the help of ETL, and then travel through the load to the back electrode. Typically, metals (such as Au and Ag), carbon, graphene, carbon nanotubes, doping, and composites are used to make the back electrode. Additionally, a circuit is completed by the initially created holes in the perovskite diffusing in the direction of the electrons leaving the load. The often-used HLT consists of p-type materials such as MoO_3_, MoO_x_, NiO, Cu_2_O, Cus, CuSCN, CuO_x_, spiro-OMeTAD, etc. [23].

### 1.3. Generations of Solar Cells

**Scheme 1:**
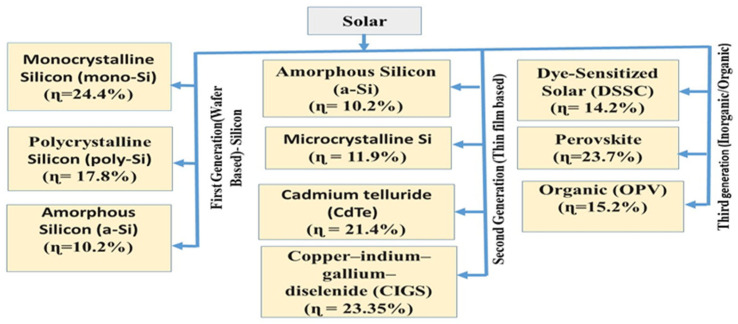
Flow diagram for the generation of solar cells [3].

Based on the nature and types of materials employed, the generations of solar cells are classified into three categories: (i) first-generation (wafer-based) solar cells; (ii) second-generation (thin film-based) solar cells; (iii) third-generation (organic or inorganic) solar cells. The overall generations of solar cells with the best efficiency record have been highlighted in the Scheme 1 [3]. The first-generation solar cells are the only ones commercially available to date, whereas the other generations are under research and development. The major advantages of first-generation solar cells are: (i) the requirement of a small installation area; (ii) a long lifecycle of over 25 years; (iii) less material waste; (iv) abundant material availability; and (v) a high heat tolerance capacity (only for amorphous silicon cells) [24]. Despite having many advantages, first-generation solar cells have a few disadvantages, such as high-cost, high temperature that lessens energy generation, kerf loss, the Staebler–Wronski effect (light-absorbing metastable fluctuations) [24], and less efficacy (for only amorphous silicon solar) [25,26]. The second-generation solar cells have several advantages, including lightweight, flexibility, cost-effectiveness, ease of fabrication, and heat bearing. However, they are less efficient and have a shorter life span [27]. Furthermore, the production of CdTe-based cells is difficult due to the toxic nature of Cd and the scarcity of telluride metal as an earth-rare element. It requires high vacuum processing as well as a time-consuming manufacturing process [24,28]. The third generation of solar cells overcomes the few problems that have appeared in the first and second generations of solar cells. The key advantages of this generation of solar cells are their widespread availability, lower processing temperature, reduced toxicity, ease of manufacturing, and ability to operate in humid and foggy conditions [24,25]. Conversely, the disadvantages of third-generation solar cells are their toxic nature, such as lead halide and ruthenium complex dyes as light absorbers for perovskites and metal complex dyes in DSSC, respectively. This generation of solar cells is also unstable for a long time and has a high rate of electron recombination. The high heat exposure breaks the materials, which lowers the solar cell’s Voc and Jsc as well as its efficiency [1,18]. Despite the numerous drawbacks of each generation of solar cells, research has continued to find ways to tweak existing materials to maintain their weaknesses. By doing this, solar research could be the turning point in reducing the world’s demand for energy.

In this review, we have discussed the variable generations of solar cells in terms of their comparative advantages and disadvantages, with an emphasis on their best efficiency. We have also included the materials used in ETLs, HTLs, perovskites, and CEs. The working mechanism of PSC and the pictorial representation involved have been discussed. Furthermore, different materials used in several components of PSC in comparison to one another’s solar parameters have been critically shown in pictorial and tabular forms in the different parts of the manuscript. Additionally, the materials used in ETL, such as SnO_2_, TiO_2_, and ZnO, their doping with other materials such as Na_2_S, KCl, MLG, etc., and a few novel materials, such as PCBM, PC_61_BM, and 2FBT2FPDI, are highlighted with their electrical and optical properties in terms of solar parameters. Similarly, the comparative study of materials that have been used for HTLs includes Spiro-OMeTAD, CuSCN, CuS, NiO, and doping such as Co-Ni and CuS-C, as well as some novel materials such as Li-TFSI and C-Co-Ni, etc. The few passivating materials and solvents that have been used to boost the performance of the solar cell and some carbon, polymeric, and nanomaterials used as CE have been highlighted in terms of their comparative performance. However, the applied materials’ comparative energy alignment profile, morphology, grain size, J-V characteristics, electrical study, and optical study have been critically discussed based on the reported results of other researchers. Finally, the current trends and commercialization possibilities have also been briefly discussed.

## 2. Interfaces of Perovskite

In the perovskite solar cell, there are various interfaces, including CE/HTL, perovskite/HTL, perovskite/ETL, and ETL/substrate [29]. The output of the device improves with the layer’s interaction level. Therefore, selective materials that might produce a stable and effective architecture can help create a better interface. A detailed discussion of the interfaces and potential materials is given below.

### 2.1. Interface of Perovskite (PSC) and HTL 

In addition to being an important component that absorbs input photons, perovskite also serves as a dividing layer by directing electrons in one direction (ETL) and holes in another (HTL). It is a crucial element that can either improve or impair the performance of a perovskite solar cell. The instability or hysteresis issue is one of the unsettling features of PSC that breaks down the current density to voltage (J-V) characteristics of the cell. The hysteresis loss can be measured during the extrinsic and intrinsic tests. Device scanning, scanning orientation (forward or reverse), and per-conditional treatment of the device, such as illumination under light or dark, are the major times when hysteresis stress develops in a device [6]. For example, copper thiocyanate (CuSCN), a p-type of semiconducting material, is incorporated into a perovskite layer in order to reduce the hysteresis loss and enhance the charge carrier mobility in the PSC/HTL layer [30,31]. Its use at the PSC/HTL interface has brought unique properties such as a wide bandgap, strong charge mobility, and a favorable valence band (−5.2 eV). Moreover, a device that included CuSCN was shown to be similarly efficient to a carbon-incorporated perovskite layer-based design based on power conversion efficiency (PCE) [32,33]. In addition to serving as a substitute for CuSCN, the carbon nanotube (CNT) served as an HTL (or bridging material) for the carbon-perovskite solar cell (C-PSC). The CNT increases the cell’s charge mobility by bridging the energy gap between the perovskite and CE and carrying the holes smoothly. Following are the benefits of using this kind of materials for future research and creation (i) long-term security, (ii) resistance to temperature, and (iii) good effectiveness [34]. Since the beginning of the PSC, spiro-OMeTAD has been a commonly used HTL. However, other innovative materials have been researched by scientists to lower the cost of the materials, improve stability and efficiency, and create an environmentally sustainable energy source. To increase the conductivity of holes, HTL uses innovative materials such as poly(triarylamine) (PTAA), lithium bis(trifluoromethanesulphonyl)imide (Li-TFSI), and its doping. However, the minor drawbacks of these HTLs, including their deliquescent, hygroscopic, and moisture-inducer properties, limit their extensive utilization in PSC. As a result, these materials may lower PCE. It may disrupt the interaction between HTL and the active layer, harm its structure, and break the electrodes through corrosion. To overcome problems related to PTAA, Li-TFSI, the carbon-based substitute materials could provide better fabrication as well as longer stability of the PSC. For example, carbonaceous materials such as graphene paste, CNTs, and fullerenes with a hydrophobic nature and better stability prevent moisture at the light-absorbing layer or the interface of perovskite/HTL [35]. Being a highly efficient HTL, Spiro-OMeTAD can also have issues, such as causing corrosion on the surface of the perovskite layer, and its high-cost challenges the cost-effective fabrication of the PSCs. The alternative materials are considered to be cost-effective as well as stable. Those alternative HTL materials are graphene paste, poly(3-hexylthiophene-2,5-diyl) (P3HT), and graphene composites [36]. The successful fabrication of perovskite solar cells can have several challenges in different interfaces, such as perovskite/ETL, perovskite/HTL, HTL/CE, ETL, and FTO. For example, the deposition of HTL materials such as spiro-OMe-TAD and CE materials such as Au coating in laboratory-based fabrication could result in waste of such materials due to improper coating techniques such as spin coating and vacuum metal deposition. These coating methods trip the solution due to the small area of the substrate or electrode, and as a result, they increase the cost of the device. For waste-free coating, other techniques could be incorporated, such as doctor blades, slot-die coating, injection printing, spray coating and screen printing, and transfer printing [37]. 

The HTL is fundamentally made of inorganic, organic, and polymer materials. Regardless of several advantages such as efficaciousness for high solar parameters, lightweight, and flexibility, these are expansive, the cell has been unestablished for a long time, and it is difficult to insert at the interface of perovskite and CE [1]. Some advanced techniques have been introduced at this interface, i.e., HTL-free perovskite. For example, the CuS (p-type semi-conductive material) incorporated with hybrid carbon CE (CuS-C) may enhance electron mobility and suppress recombination at the perovskite and CE boundaries. The hole conductivity of CuS-C is higher, which provides a fast rate of charge transportation from the light-absorbing layer to the external circuit. It was found that the hybrid HTL was more efficient than the HTL-free PSC. The efficiencies for CuS-C and pure carbon PSC are 11.28% and 10.27%, respectively [2]. Several techniques have been used to improve the hole conductivity at the interfaces of perovskite and CE. In this regard, Xie et al. [38] reported Co-Ni-doped carbon aerogels with an unalike ratio of cobalt and nickel metals. The Co-Ni-C-based aerogel provided a core-shell structure, which also exposed noble constancy in an acidic solution. The solution was uniformly inserted between the perovskite and CE interfaces. This demonstrated a PEC of 12.05%. This cell architecture was found to be superior for increasing the linkage between the layers, hole conductivity, and stability for long time intervals. Similarly, NiS-C (nickel sulfide carbon composite) was introduced as the HTL [39]. Gao et al. reported bio-carbon-based low-cost HTL, which acts as CE too, and the highest PCE for this cell was found to be 12.82% [16]. Meng et al. determined the efficiency of the cell and the ultra-low-cost HTL, which also functions as CE, to be 10.87% [40]. The NiO nanoparticles were used at different concentrations (20 mg mL^−1^, 25 mg mL^−1^, and 30 mg mL^−1^) as HTLs by Cai et al. [41] to examine the effectiveness of the cell without HTL. The greatest efficiency of the NiO HTL-based cells was 13.6%, which was discovered to be more efficient than cells without HTL. The hole transporting material (HTL) is essential for increasing the solar cell’s efficiency and lowering the cost of the cells. Researchers are now concentrating on HTL-free PSC as well as combining HTL with CE to reduce thickness and material costs. As an illustration, Geng et al. [39] created a composite structure of C-Co-Ni with varying weight ratios of Co-Ni (0.5%, 1.0%, and 3.0%) and compared the efficiency of the cells with commercially available conductive carbon (CC). The highest efficiency of the cell with 3% (*w*/*w*) was 10.22%, proving that the efficiency of composites is higher than CC [42]. Furthermore, one of the novel approaches is dopant-free HTL, which has a high ability to enhance solar performance by using thiazolothiazole-thiophene-based polymers. The polymer has good conductivity, stability, and crystallinity. The best efficiency of the cell incorporated with this polymer provided 14.02% and retained 80% power conversion when the cell was kept in N_2_-atmosphere over 113 days [43]. Similarly, the two HTL materials (poly (3-hexylthiophene-2,5-diyl) (P3HT) and poly [bis (4-phenyl) (2,4,6-trimethylphenyl) amine] (PTAA)) have been compared in terms of stability. Both material-based HTLs were found to be transparent and showed better conductivity and compatibility with the perovskite layer. Among these two HTLs, P3HT was more stable than PTAA [44]. However, as the perovskite/HTL interface is one of the key paths to the flow hole, better interaction between these two materials is crucial for better solar performance. For better interaction, the band gap energies of these two materials must match at the HOMO level. The selection of the material, concertation of the HTL solution, and proper deposition method are considered important factors that may boost the performance of PSC.

### 2.2. Interface of ETL and Perovskite 

The ETL is the major component of PSC that carries the electrons obtained from the active layer to the substrate in a planar structure and to the CE in an inverted structure. The interface between the ETL and perovskite layer is one of the major sites in the PSC, which affects the performance of the entire cell. In ETL, the most useful materials are mesoporous titanium oxide (m-TiO_2_), compact titanium oxide (c-TiO_2_), zinc oxide (ZnO), Al_2_O_3_, SnO_2_, m-ZrO_2_, PEDOT:PSS, carbon materials such as C_60_, doping of organic and inorganic materials, etc. These are wide bandgap nanocrystalline semiconductors [45,46]. These semiconducting nanocrystals used in this layer are n-type semiconductors, and they work as electron transporter to collect the charge in the light-absorbing layer. Among the semiconductor nanocrystals, TiO_2_ as an ETL has been achieving the best efficiency to date at 21.25% [47], but the main issue with TiO_2_ is the requirement of high annealing temperature to grow the crystal on the substrate. Generally, the required temperature for the deposition of TiO_2_ is above 400 °C. The relatively less temperature-based material (for example, ZnO) can overcome such challenges. The ZnO NPs require a temperature of around 100–300 °C for depositing on the substrate, but annealing temperature is not the only issue because a lower annealing temperature may result in weak crystallinity and surface morphology. In order to enhance morphology, crystallinity, and ETL’s interaction with perovskite, several precursor solutions have been applied to the fabrication of perovskite solar cells [48]. The comparative ratio of solvent, doping of the materials with variable ratio, novel passivate with their direction of scan, effect layers’ thickness, and effect of annealing temperature in comparison to the four solar parameters are illustrated in Table 1. 

The efficiency and open circuit voltage (Voc) of the two types of devices (the first using pure SnO_2_ as ETL and the second using 1% weight of TaCl_5_ doped with SnO_2_ as ETL) were compared. The efficiency and Voc of the doped cell were found to be slightly higher than those of the non-doped cell. The cell efficiency and Voc of the non-doped and doped cells were 16.39%, 970 mV, and 18.23%, 1080 mV, respectively [55]. To fabricate the solar cells, aluminum zinc oxide (AZO) nanoparticles were used as the ETL, and phenyl-C_61_-butyric acid methyl ester (PCBM) was placed between the AZO and perovskite layers. Additionally, the performances of the cells were compared, where the cell incorporated PCBM provided about 14% higher efficiency than AZO ETL. The solar parameters such as Voc, Jsc, FF, and η for the device with AZO were 1120 mV, 16.3 mA cm^−2^, 71%, and 12.9%, whereas, for AZO/PCBM, they were 1120 mV, 20.1 mA cm^−2^, 75%, and 16.9%, respectively. Among the devices, the PCBM-embedded layer-based device provided better efficiency because the layer did not disturb the original properties of the light-absorbing layer, enhanced charge mobility, and helped in better interaction [48]. As a low temperature-based common ETL, PCBM can have some disadvantages, i.e., expensive, has a different energy level than perovskite (energy incompatibility), and breaks down while contacting light for a long time [48]. Although this layer may provide less light exposure to the active layer, it leads to improper interlinking and bulk charge carrier recombination at perovskite. A novel PSC fabrication includes a small organic material, Perylene Diimide dimer (2FBT2FPDI). It is an n-type precursor kept in between MAPbI_3_ and PCBM in order to passivate the surface defect between the perovskite and ETL [39]. The thickness of the layer is one of the important aspects that influence the performance of the solar cells. For example, the solar cells were fabricated with the different thicknesses of 2FBT2FPDI precursor (7 nm, 13 nm, and 21 nm) and PCBM (control) [52]. Among the various thickness passivation layers, the 13 nm layer provided the highest efficiency of 20.3%, which is significantly higher efficiency than other thicknesses of the 2FBT2FPDI and PCBM (16.8% and 17.3%, respectively). This organic material may have provided better efficiency due to the better conjugation with lead molecules, the repair of the surface defect of the layer, and a reduction in the stocked electron recombination rate [52]. The overall solar parameters are listed in Table 1. Modifying the electron transporting materials in order to enhance the performance of the cell employed the two ETLs: (i) TiO_2_ nanograss (NG), and (ii) 2D TiS_2_−TiO_2_ NG. The photovoltaic performance of both of the cells was compared, and 2D TiS_2_−TiO_2_ NG revealed a slightly higher performance than that of TiO_2_ NG. This higher performance of the cell containing 2D TiS_2_−TiO_2_ NG as a passivating layer is due to the fast charge carrier mobility, which reduces the stocked charge trapping density, and increases the stability of the cell. The solar parameters for a 2D TiS_2_−TiO_2_ NG-based cell are 1150 mV (Voc), 22.40 mA cm^−2^ (Jsc), 76% FF, and 18.73% efficiency, whereas they are 1140 mV (Voc), 21.90 mA cm^−2^ (Jsc), 76% (FF), and 18.14% efficiency, respectively for TiO_2_ NG [56]. The PSCs were prepared by using SnO_2_ and 20% Eu doped in SnO_2_ as ETLs, and the efficiencies and stabilities (in an N_2_ atmosphere with high temperature, darkness, and high humidity) were compared for both cells. Both of the cells were illuminated under a 40–50% moisture environment at 60 °C for 300 min. Moreover, the cell doped with 20% Eu retained 87% of its initial efficiency above 84 days, while the cell containing SnO_2_ as ETL dropped 50% of its initial efficiency, making the superior cell’s efficiency 20.14%. The superior PEC of SnO_2_:20% Eu is due to the doped structure containing the Eu ion, which has a better ability to passivate the surface defect between the ETL and perovskite interface. It also adjusts the balanced humidity atmosphere at that interface and enhances the environmental solidity of the cell [57].

Another novel solar cell consisting of four types of fullerene derivatives tested for the ETL: (i) PC_61_ BM; (ii) BPy-C_60;_ (iii) BpAn-C_60_; and (iv) BAn-C_60_. The PC_61_-BM precursor material was kept as a control, while the remaining three were taken as variables for the PSCs. When the PCE of the three cells were compared with the control, the highest PEC was found for the control, while the remaining cells’ efficiencies declined. The PCE values for the cells PC_61_ BM, BPy-C_60_, BpAn-C_60_, and BAn-C_60_ were 15.43%, 14.59%, 12.92%, and 3.42%, respectively [41]. As an ETL, these devices have provided average performance, but the same fullerene derivatives have been employed at the interface of ETL and perovskite with variable thicknesses (3 nm, 5 nm, 10 nm, and 20 nm) with C_60_ as an HTL, which significantly enhanced the performances. The overall thickness of the cell was 85 nm. Although the highest efficiency of the cell employed BPy-C_60_ (the electron-exciting layer) in the interface between perovskites, the efficiency was 18.88% with a layer thickness of 3 nm. The enormous efficiency enhancement for the cell is due to the main linking groups, such as pyridine, carbonyl, and carbamate, and the Pb^+2^ ions present in the perovskite. Thus, this interfacial material may have increased the surface conductivity of the layer and bridged the gap between ETL/perovskite [58]. Guo et al. used two types of ETL, namely SnO_2_ and SnO_2_:InCl_3_ (tin oxide doped indium tri-chloride) and compared both of the cells in terms of two solar parameters, Jsc and η. The values of Jsc and η were found to be raised for doping (22.1 to 23.7 mAcm^−2^) and for SnO_2_ (19.1 to 20.8%), respectively, for planar PSCs. Consequently, the SnO_2_:InCl_3_ had a better passivating effect because of its lower film thickness, higher grain density, and faster carrier movement. In addition, Cl is considered a better passivating material or fast carrier transporter due to its bonding ability with Pb present in the light absorbing layer, i.e., Pb-Cl. Furthermore, indium may have better doping properties as it has less reactivity and better corrosion resistance [21]. 

Additionally, several other factors could hinder the performance of the perovskite solar cells; those could be in the interface or external atmosphere. Factors such as moisture, temperature, oxygen, and ultraviolet radiation mainly degrade the device’s performance [59]. The ultraviolet radiation hinders the cell’s performance by inserting a fault in TiO_2_ through the catalysis of Ti^3+^. As a result, it imbalances the performance of ETL as well as the morphology of the cell. To overcome this UV effect, polyethyleneimine ethoxylated (PEIE) material needs to be incorporated between ETL and perovskite. This PEIE can reduce the optical stress in semiconducting nanomaterials, i.e., TiO_2_, by blocking Ti^3+^. As a result, this may increase the performance of the cells. However, the PEIE material may form the poor crystal growth of perovskite due to the surface roughness of PEIE and can compose the island of the particles [59]. E.g., the nanocomposite structure of tellurophene (2-D metal-organic framework, MOF) and PEIE showed strong interaction due to the amine group present on the PEIE and metal ions in the MOF. This PEIE and metal-organic exfoliation can replace the electronic cloud present on the surface of TiO_2_ nanoparticles that was created by ultraviolet radiation. This TiO_2_ and MOF not only enhance the power conversion efficiency but may also help expand the damage caused by PEIE on the surface of the perovskite. Additionally, this may increase the stability of the device. Thus, the efficiencies of the cells containing: (i) TiO_2_/perovskite, (ii) TiO_2_/PEIE/perovskite, and (iii) PEIE-2D MOF/TiO_2_/perovskite were compared. The efficiency of the first cell with only TiO_2_ ETL was slightly higher than the PEIE-incorporated ETL, but the efficiency of the PEIE-based 2D MOF TiO_2_-based passivating layer was significantly increased than that of TiO_2_-based ETL. The efficiencies of the cells based on the integrated ETLs such as TiO_2_, PEIE/TiO_2_, and PEIT-2D MOF TiO_2_ were 20.42%, 19.75%, and 22.22%, respectively [59]. Two types of ETLs were introduced: the first cell used SnO_2_ ETL (spin coating in a nitrogen atmosphere), and the second used a colloidal mixture of SnO_2_ and phosphoric acid [4]. The solar parameters for cells containing SnO_2_ as ETL were Voc 1180 mV, Jsc 22.54 mAcm^−2^, FF 73.81%, and efficiency 19.67%, whereas, for SnO_2_ phosphoric acid precursors containing ETL, they were 1170 mV Voc, 23.20 mAcm^−2^, 77.40% FF, and 21.02% efficiency. This dramatic enhancement of the efficiency of the cell with SnO_2_ phosphoric acid precursor in comparison to pristine SnO_2_ ETL is due to the following two reasons: (i) the phosphate group makes strong binding with SnO_2_ nanocrystals (i.e., Sn-O-P), which reduces the potential hurdle; and (ii) it makes a smooth passivating layer at ETL and perovskite. The concentration of the phosphoric acid solution used to make the precursor was 7.4% by volume [4]. A novel technique called atomic layer deposition (ADL) involves the low-temperature deposition of a precursor at the interface of ETL and perovskite. The ADL has been used for the deposition of tetrakis-dimethyl-amine-tin (TDMASn) for the PSC. It was found that TDMASn-based ETL provides better efficiency for the PSC, i.e., 20%. This ADL has several advantages for the deposited film, such as enhancement of electrical, optical, and chemical properties and better interfacial passivation at low temperatures [60].

The PCBM was used as an ETL; it works as a stable electron-collecting layer as well as a defense against photon-based degradation and oxygen resistance. This PCBM film acts as a barrier, or seal, for oxygen on this surface as well as a quencher of superoxide. The quenching effect of PCBM mainly depends on the electron affinity and LUMO (Lowest unoccupied molecular orbital). The LUMO is responsible for improving the open circuit voltage and increasing the vulnerability to superoxide-facilitated degradation [61]. Likewise, the cells were fabricated using Na_2_S doped TiO_2_ semiconductor nanocrystals with various ratios (0.5%, 1%, 2%, and 3%), TiO_2_ doped with 1% NaCl, and pristine TiO_2_ as ETLs. All the cells were found to be efficient. Among different concentrations of Na_2_S doped in TiO_2_, the cell with 1% provided the maximum efficiency of 21.25%, which is higher than the other percentages of doping, pristine, and TiO_2_ doped in 1% NaCl. With this data, it is clear that doping is a superior technique to enhance the PCE of the solar cells. In ETL, the dopant containing Na^+^ and S_2_^-^ plays a key role in improving surface passivation. Na^+^ ions are mainly responsible for enhancing the conductivity at the interface as well as fast electron injection from the TiO_2_ to the FTO, thereby improving the Jsc and FF of the device. On the other hand, S_2_^-^ ions are responsible for creating the hydrophobicity at the surface of TiO_2_ as well as being responsible for many other factors such as (i) making a bond with perovskite (S-Pb), which then increases the ETL/perovskite interaction, (ii) increasing the crystallinity of perovskite then it reduces the charge trapping and increase the grain size, and (iii) increase the stability of the cell [47]. Three types of ETLs have been reported in the literature [54]. They have included ZnO and mono-layer graphite (MLG)/ZnO as ETLs, and MLG/ZnO with passivation for planar PSC [54]. All the cells were efficient in both forward and reverse directions. However, the backward scanning provided more efficiency than the forward direction. In addition, the cell with MLG/ZnO as the passivating layer provided the highest efficiency compared to the MLG/ZnO and pristine ZnO-based ETL. The efficiencies for ZnO, (MLG)/ZnO as ETLs, and MLG/ZnO passivating layers were 13.82%, 19.81%, and 21.03%, respectively [54,62]. The overall solar parameters are shown in Table 1. The novel carbonaceous ETL material called penta-diamond is considered one of the unique materials due to its n-type indirect bandgap semiconductor (bandgap of 2.46 eV) and the energy level of this material being comparable to the common sorts of carbon materials such as PCBM. Hence, the penta-diamond as ETL may enhance electronic movement toward the substrate and reduce interfacial electronic recombination [63]. Thus, doping with variable ratios, novel materials and their doping with commonly used ETL such as TiO_2_, SnO_2,_ etc., and favorable sintering temperatures, solvents, thickness, and effective passivation are the main strategies that can have a significant impact on the enhancement of the performance of PSC. 

### 2.3. Quality of the Light Absorbing Layer for Stable and Efficient Perovskite Solar Cells

The perovskite is an active layer that is responsible for harvesting the incident light. A perovskite solar needs a better quality light-absorbing layer to produce high current and voltage. Although the higher-quality light-absorbing layer helps to reduce carrier recombination and enhances charge mobility and stability over long time intervals, several aspects have been incorporated to enhance the efficiency and stability of the PSCs. For example, to improve the quality of the light-absorbing layer, the crystal structure should be controlled. The crystal structure can be controlled by adding (i) butylphosphonic acid and 4-ammonium chloride to the perovskite solution, and (ii) 1,8-diiodooctane to the perovskite solution [64]. Zhang et al. [65] incorporated two types of precursor solutions into the perovskite (MAPbI_3_) solution. They prepared three types of free hole-conductive devices (i) MAPbI_3_ (control); (ii) ZnAc_2_ solution added into MAPbI_3_; and (iii) NH_4_Ac solution added into MAPbI_3_. Among these two additives, NH_4_Ac was more effective in improving crystallinity compared to ZnAc_2_. For these devices, optical characteristics such as lower defect density and lower charge recombination speed may result in better power conversion efficiency. However, the device with ZnAc_2_ additive provided higher optical absorbance and smaller series resistance, whereas the device with pure perovskite and NH_4_Ac additive provided better air stability. The PECs for pure MAPbI_3_, MAPbI_3_: ZnAc_2_, and MAPbI_3_: ZnAc_2_ were 9.79%, 11.34%, and 13.15%, respectively [65].

The light-harvesting layer (perovskite) was treated by a 1,3-dimethyl-3-imidazolium hexafluorophosphate (DMIMPF_6_)-based precursor solution at the interface of the HTL side of perovskite [66]. This is an ionic liquid (IL) solution that has several characteristics that are listed as follows: (i) successively modify the energy alignment between perovskite and HTL, (ii) a high tendency to passivate the light-absorbing layer due to the establishment of electrostatic interaction between Pb^2+^ ions present in the light-absorbing layer, and (iii) protection against oxygen and humidity due to its hydrophobic nature. Therefore, the passivation of perovskite by DMIMPF_6_ can have a significant effect on the efficiency of PSC. For example, the devices with and without DMIMPF_6_ treatment were compared in terms of solar efficiency. The device treated with the DMIMPF_6_ solution was found to be more efficient than one not treated with the solvent. The power conversion efficiencies for cells with and without DMIMPF_6_ were 23.25% and 21.09%, respectively [66]. A novel method called nanocrystalline pinning was used by Wang et al. In this method, the perovskite was passivated by a 2-bromo ethyl-tri-methyl ammonium bromide (BETAB) nanocrystal as follows: (i) BETAB was added into the perovskite; (ii) preparation of BETAB colloidal solution by self-assembling in isopropanol; and (iii) identification of the major compound present in BETAB, where the major compound is CH_2_CH_2_Br [64]. This passivation was tested for the fabrication of two cells with and without BETAB, where the cell consisting of BETAB as a passivating layer was found to be superior to the cell without BETAB in terms of solar parameters. The magnificent features of BETAB over the cell without BETAB are: (i) it conquers the formation of the pb(0) the state of pb present in perovskite, (ii) it passivates the vacant sites present in iodine, (iii) it creates a pb-I anti-site defect mechanism in perovskite film. This passivation enhanced the carrier’s lifetime of electrons and holes, Voc (1.14 V), and reduced non-radiative recombination. Thus, BETAB-based solar cells have characteristics such as thermal solidity, moisture protection, and stability for a long time. The highest efficiency for the cell with BETAB passivation was found to be 23.04% for the planar PSC device [67]. The stability of the perovskite passivating cell and non-passivating cells are shown in Table 2. 

The planar PSCs based on the FA_0.85_MA_0.15_ PbI_3_ light-absorbing layer were prepared by Quan et al. [72] using a polyfluoroorganic compound, i.e., tris(pentafluorophenyl)boron (TPFPB), as a surface passivating layer at the interface of HTL and perovskite [72]. The efficiencies of the passivating and non-passivating cells were compared. The efficiencies for the cells consisting of TPFPB and without TPFPB as a passivating layer were 19.55% and 21.60%, respectively. The overall performance of the cell containing TPFPB was better than that of the cell without TPFPB. From this study, the authors conclude that the TPFPB is a promising technique not only for improving the PCE of the solar cell but also persuasively for growing quality crystal, reducing passivation failure, increasing electron injection, air stability, light stability, and hydrophobicity [72]. Another novel passivating technique is organic halide salts (4-fluorophenethylammonium iodide, F-PEAI) [68]. This layer was incorporated directly on top of the perovskite at a concentration of 2.5 mg/mL. The solar parameters were compared with those of the cells fabricated without any passivating materials. It was found that the cell that engaged F-PEAI as a passivate provided over 4% more efficiency than the cell without F-PEAI. However, the efficiencies for the cells with and without F-PEAI were 21% and 19.5%, respectively. Both cells were tested at 60 °C in humid conditions for 720 h, and the initial stabilities for cells with and without F-PEAI were calculated to be 98.9% and 93.3%, respectively. After the given time interval, the cells containing F-PEAI retained around 90% of their stability, while those without F-PEAI retained only 70%. From this study, it is clear that F-PEAI is one of the promising materials for fabricating stable PSC [68]. A different novel passivating small molecule called indacenodithieno[3,2-b]thiophene (0.3%wt IDTT-ThCz) was employed on top of the perovskite [69]. When the cell’s performance with this precursor solution and the pristine cell’s performance is compared, it is found that the efficiency of the cell consisting of IDTT-ThCz perovskite passivation provided more efficiency than the pristine cell (20.3 and 22.5%, respectively). The higher efficiency, as well as other solar parameters, were observed for the cell containing IDTT-ThCz due to the interaction between the thiophene group present in the core and the end-capping group at the peripheral of IDTT-ThCz with PbI2 of perovskite. This passivation has improved charge mobility and reduced the interface defect and perovskite degradation. As a result, this passivation layer has also demonstrated good stability at 80 °C humidity for 500 h [69]. The overall initial stability and the final stability are shown in Table 2.

In addition, a fluorinated organic ammonium halide salt or 4-trifluoromethyl phenethyl ammonium iodide (CFPEAI) precursor material was used as a perovskite passivating layer for the CsPbI_2_Br-based light-absorbing layer. Another solar cell was also prepared without passivation to observe the efficiency’s growth or decrease. The cell fabricated without a passivating layer and with CFPEAI provided efficient solar energy, and the efficiencies are 14.50% and 16.07%, respectively. The encapsulation of CFPEAI-based cells was superior because it reduced non-radiative recombination of the cell, hysteresis loss, and the solidity of the electronic cloud. Overall, it had a hydrophobic nature, which enhanced the stability of the solar cell, and it could be one of the unique techniques to improve the efficiency of the solar cell [73]. To incorporate the PEAI as a surface passivating layer in between perovskite and HTL (Spiro-OMeTAD), the efficiency of the cell was compared with that of a solar cell consisting only of Spiro-OMeTAD as HTL [70]. It was found that the PEAI-based cell was less efficient than the spiro-OMeTAD despite delivering reasonable stability and efficiency. This study reveals that all of the passivating materials are not considered effective in terms of solar performance, but they may reduce the cost of the cell as well as provide flexible architecture [70]. Passivation is one of the key strategies for the PSCs in order to enhance the cell’s performance, stability, cost-effectiveness, and flexibility. The passivating layer is named 1-Ethylpyridinium Chloride (EPC), which is a small organic ionic compound in between the HTL and perovskite. Although the efficiency of the cells with and without EPC was compared, the cells delivered efficiencies of 19.52% and 21.19%, respectively. The dramatic improvements in the efficiency of cells containing EPC are due to the following reasons: (i) the pyridine group has a strong interaction with lead ions, which may reduce the defect’s compactness, (ii) the pyridine and ethyl functional groups conquer the moisture and make it hydrophobic, and (iii) chlorine improves the passivation and enhances the crystallinity of perovskite. Therefore, it enhances the electrons’ and holes’ mobility and improves stability and efficiency [74]. Ezike et al. [75] reported the use of a hydrophobic material, i.e., 4-tert-butylpyridine (tBP), for the passivation of the perovskite layer. The passivated layer’s performance was tested for 8 h under the light. Similarly, the solar cell without tBP was also observed for the same time interval. The efficiencies with and without tBP were found to be 10.32% and 6.37%, respectively, at 0 h. Although the cells with the same state as their initial condition were tested at 8 h, the efficiencies with and without tBP were found to be 9.35% and 1.66%, respectively. Consequently, this study shows that the solar cells passivated with some materials can have better stability and efficiency than those not passivated. This tBP has shown significant potential against moisture and air and is considered a hydrophobic material for PSCs. CF_3_PEAI-based 2D passivation was found effective for the efficient solar cell, with better hydrophobic properties, reduced electronic trapping clouds, and enhanced electronic mobility [76]. The overall stability at a 70–80% humidity level is mentioned in Table 2. The poly[(9,9-dioctylfluorenyl 2,7-diyl)-alt-co-(1,4-benzo-{2,1′,3}-thiadiazole)] (PFBT) with different concentrations (1, 2.5, 4, and 5 ppm) incorporated in between the perovskite and HTL interface [77]. It was found that the cell with a concentration of 2.5 had the best efficiency compared to the control and the rest of the concentrations. At this concertation, the efficiency was found to be 17.21%. This precursor material has revealed improvements in (i) passivating failings, (ii) crystallinity, (iii) conquering the nonradioactive recombination of electrons, and (iv) reducing the charge cloud at this surface [77]. Krbyk et al. [78] reported nitrogen-doped CQDs with different concentrations (1, 3, and 5%) mixed with the perovskite solution. The cells without any doping and those with concentration-based doping were compared in terms of efficiency. It was found that the cell with a 3% concentration of doping produced the highest efficiency than the rest of the cell, i.e., 13.93%. This technique was found to be optimistic towards solar cell performance due to the increase in gain size (the largest grain size for 5% N-CQDs), strong binding of carbonyl and amine with uncoordinated Pb at the light-absorbing layer, reduction of the passivating defect, and non-radiative recombination-based loss. A unique passivation method included the passivation of both sides of the charge transporting layer, i.e., ETL (perovskite) and HTL (perovskite). This double-sided 3D PSC-based bulky organic cation (n-BAI or n-butylammonium iodide) has been found to be optimistically layer passivated. It has produced several superior characteristics to one-side passivation, including reduced interface base charge recombination and the maintenance of band gap energy alignment at the interface of perovskite/ETL and perovskite/HTL. It enables the uniform passivation of both sides of the light-absorbing layer and, as a result, improves the solar parameters such as (Voc) 1200 mV, (Jsc) 23.97 mA cm^−2^, (FF) 79.31%, and (η) 22.77%, which are higher than one-sided passivation as well as non-passivated solar cells [79]. Another similar (mixed-dimensional 2D–3D PSCs) method has included n-BAI, iso-butylammonium iodide (i-BAI), and DIAC for the passivation of both sides of the light-harvesting layer [80]. Herein, the two isomers of butyl ammonium and DIAC have provided higher efficiency than the control. The extracted efficiencies for n-BAI were 22.50%, i-BAI was 22.23%, DIAC was 23.27%, and the one for the control cell was 20.94%. This mixed 2D and 3D layer has improved the passivation weakness and electron recombination between the ETL and HTL and grown the crystallinity of perovskite. As a result, it has calibrated the stability and enhanced the efficiency of the PSCs [80]. To incorporate the passivation layer at the interface of ETL and perovskite in order to improve the passivation defect, reduce electron recombination at the interface, and reduce the non-radiative electronic loss. The three different concentrations of silicon oxide (SiO_x_–0.5, SiO_x_–1, and SiO_x_–2%) incorporated with TiO_2_, then passivate the ETL side (at the interface of ETL and perovskite). It was found that silicon oxide with 1% concentration mixed with TiO_2_ on perovskite provided better efficiencies than the other concentrations. The highest efficiency of the cell at 1% concentration was 18% [81]. Solvent vapor annealing (SVA) can be considered one of the advanced methods to improve the perovskite crystallinity and morphology. In this method, when the active layer is getting ready, then during the annealing phase, it is immersed in the solvent vapor environment. SAV basically contains polar solvents such as N, N-dimethylformamide (DMF), or dimethyl sulfoxide (DMSO). The best efficacy obtained from the cell-optimizing SAV is 18.51% [82].

### 2.4. Effect of the Carbonaceous Counter Electrode (CE) on the Performance of Perovskite Solar Cells

The counter electrode or back electrode can have an indispensable role in completing the circuit for the successful transfer of electrons and holes in a perovskite solar cell. Initially, Au-based CEs were widely applied for the PSCs due to their high conductivities [12,18], but in the past few years, carbon-based novel nanomaterials and non-carbonaceous nanomaterials as CEs have gained attention for the PSCs fabrication [5,83]. The commercial carbon paste was used as a CE for the solar cell that was coated on top of the HTL by the doctor-blade method and then heated at 120 °C for 20 min [84]. The efficiency of this CE solar cell was 11.21%, which does not compete with the Au-based CE but certainly provides a cost-effective PSC [84]. Cai et al. [41] coated commercial carbon paste directly on the perovskite layer by doctor blade method and heated it at 80 °C for 30 min (layer thickness: 20 mm). The cell’s highest efficiency was found to be 13.6%. The scaffold-based CE was coated with commercially available carbon paste with the help of the doctor blade coating technique on top of the perovskite [5]. One of the unique methods that altered the position of HTL and CE gave the following solar structures: (i) FTO/SnO_2_/Cs_0.05_ FA_0.83_ MA_0.17_ PbI_2.53_ Br_0.47_/CuSCN/C_,_ and (ii) FTO/SnO_2_/Cs_0.05_ FA_0.83_ MA_0.17_ PbI_2.53_ Br_0.47_/C-CuSCN. The carbon paste was used as a CE by the doctor blade method when coating the first device. The carbon paste was mixed with the HTL for the second device. For the third device, the carbon paste was used as both HTL and CE [6]. It was found that the solar cell containing C-CuSCN as HTL and CE provided the best efficiency, which was 14.8%. The incorporation of carbon paste has gained attention for the new research due to: (i) its reduction of hysteresis loss; (ii) its reduction of charge recombination at the interface; (iii) its enhancement of the charge carrier’s mobility; and (iv) its resistance against moist conditions and long-term stability [6]. Another study included graphene-based CE for two sorts of solar cells, planar and mesoscopic. The planar solar has a slightly higher efficiency than the mesoscopic. The efficiency for planar and mesoscopic solar cells is 14.06% and 13.85%, respectively. These graphene-based CEs, in terms of cell efficiencies, were found to be better than Au CE. Thus, these C-CEs may gain attention in future studies due to their low temperature-based fabrication, low hysteresis loss, and relative cost-effectiveness compared to Au-based CEs [36]. For the fabrication of CE, 180 L of chlorobenzene containing 1 mg/mL of single-walled carbon nanotube (SWCNT) was dripped onto the substrate, which was then heated at 100 °C for 60 min [34]. Carbon composite was added on the top of the SWCNT-coated layer by the doctor blading technique, and the same heat was supplied as SWCNT. In addition, for the other CEs, they have incorporated three different graphites: flaky graphite of 12,500 mesh (CE A), 8 µm spheroidal graphite (CE B), and flaky graphite of 15,000 mesh (CE C) on carbon black in the ratio 1:3. Thus, the relative efficiencies for the cells such as CE A, CE B, CE C, and MWCNT-based composites were 11.72%, 12.63%, 13.86%, and 15.73%, respectively. This carbon nanotube-based bridging technique has superior characteristics over fast charge creation, better movement, and better interfacial activity. These solar cells were found to be stable in a 75 °C humid environment for more than 90 days. Due to such superior reasons, this technique can be considered effective for future research on perovskite solar cells [34]. The HTL-free carbon CE-based PSC was found efficient (=5.86% and Voc = 1340 mV) and stable at 50–60% humidity for 240 h [85].

In an HTL-free C-CE-based perovskite solar, α-CsPbI_3_ was incorporated as a light-harvesting layer. The cell containing α-CsPbI_3_ as an active layer was found stable (80 °C and humidity) and efficient (η = 9.5%) for over 3000 h and retained 90% of its initial stability [22]. The PSC architecture, which incorporates FTO, TiO_2_, and CsPbI_2_Br (carbon-based composition), has one of the highest C-CE efficiencies (14.84%). It has also given better stability, i.e., it has retained 94% of the initial stability over 30 days at ambient air conditioning [35]. Similarly, in a C-CE -based cell containing tungsten oxide carbon composite (FTO/c-TiO_2_/m-TiO_2_/m-Al_2_O_3_/MAPbI_3_/WO_3_-carbon), stability was tested for approximately 500 h in ambient air in the presence of light. The cell has reserved about 85% of the initial efficacy (η = 10.3%) at the given condition. The cross-stacked carbon nanotube (CSCNT)-based CE used for the planar inorganic PSC provided an efficiency of 11.9%. Again, this material was applied as CE with spiro-OMeTAD HTL and provided 8.4% efficiency. Additionally, for the same cell, all the carbon materials are incorporated in ETL (PCBM with TiO_2_) and CE. This is the only carbon-based flexible solar cell that has retained around 90% of its initial efficiency for 1000 h in the presence of light in a humid environment. Due to its long-term stability, efficacy, cost-effectiveness, and flexibility, this technique can be considered a promising fabrication procedure for future research and development [7,86]. Furthermore, the HTL-free CE was prepared for the cost-effective PSCs with the help of spin coating (at ambient air and low temperature). This technique has several benefits, such as less material waste, adaptability, consistency, and unique design. The spin coating was compared with the doctor-balding coating in terms of stability and efficiency. The solar cells coated with spin coating and doctor blading have efficiencies of 14.41% and 11.14% (for ambient air and low temperature), respectively. Therefore, spin coating is considered one of the most cost-effective, flexible, and unique methods for the fabrication of perovskite [37]. The hydride-structured carbon CE provided the best stability and protection, retaining 90% of its initial efficacy (=11.28%) for 700 h [2]. Meng et al. [40] reported coal-based carbon energy (CE), which was stable at 30% humidity for 120 h and 10.87% efficiency.

Jiang et al. [87,88] reported the fully printable CE for the mesoporous perovskite solar cell at a low temperature (50 °C). This device has delivered reasonable efficiency, i.e., 14.04%. Some of the positive aspects of this device are sufficient conductivity at a low temperature, cost-effectiveness, and ease of fabrication. Y. Zhong et al. [89] reported two types of CE: needle coke (NC) and carbon black (CB). They compared the solar calls in terms of solar parameters and found that the NC-based cell has provided superior (Voc) 890 mV, (Jsc) 23.63 mA cm^−2^, (FF) 55.56%, and (η) 11.66%. Similarly, for CB-based cells: (Voc) 830 mV, (Jsc) 19.17 mA cm^−2^, (FF) 47.36%, and (η) 7.49% were recorded. The higher efficiency of the NC CE is due to its fibrous composite structure and finer pore size than CB, the filling of perovskite occurring faster in NC, increased charge transport mobility, reduced hysteresis loss, and better conductivity than the CB CE. Gao et al. [10] reported that the biomaterials extracted carbon materials prepared from corn stalks (CS-B), peanut shells (PS-B), phragmites australis (PA-B), and bamboo chopsticks (BC-B). Herein, these bio-carbon-based materials were applied for HTL-free CEs. Among these waste-based biodegradable materials, BC-B was found to be the most efficient than other materials. The efficiencies for the solar cells with the CS-B, PS-B, PA-B, and BC-B were 6.36%, 7.85%, 10.30%, and 12.82%, respectively. Similarly, Liu et al. [90] prepared CE with PCE 12.05% from the soybean dregs-based bio-carbon. This efficiency remained constant after inspection at 960 h in the air. However, these biomaterial-fabricated solar cells are found to be reasonably effective due to their fast rate of transportation of charge carriers, optimum bandgap, minor radiative recombination loss, and cost efficiency [15]. Meng et al. [40] reported the ultrathin coal-based PSC. This seamless electrode was treated with an anti-solvent called acetylene black in several concentrations (0, 10, 20, and 30 mg/mL). It was found that the solar cell treated with 20 mg/mL provided the highest efficiency than the other concentrations of antisolvent-based treatment. As coal is the most widely available and cost-effective material, it also provides better stability for the solar cell at 30% humidity for 120 h [41]. In addition, the conductive carbon paste CE with 9.45% efficiency was introduced by Gong et al. [91]. The mesoscopic solar employed C-CE, i.e., the cell structure FTO/TiO_2_/CH_3_NH_3_PbI_3_/NiO/C, as reported by Cai et al. [41]. The efficiency of the cell was found to be 13.6%. The combination of nano-carbon (carbon black) with graphite is one of the novel techniques to enhance the stability and efficiency of the HTL-free solar cell. This device has specified humidity-based protection due to the hydrophobic carbon film coated on top. It may have the potential for larger-scale fabrication. The best efficiency for the solar cell inspected after 360 h was 9.5% [92]. Three types of carbonaceous materials, such as carbon black (CB), graphene sheet (GS), and graphene (G), were used for the CEs. The solar parameters have also been compared, and overall, the cells were found to be efficient. The increasing order of efficiency of the solar cells was CB < GS < G, and overall parameters are listed in Table 3.

Among these three carbon-based cells, graphene was found to be superior in terms of overall parameters. This may be to reduce energy damage during the charge carrier collection through the carbon CEs. In addition, G has provided the advantage over moisture and heat for over 500 h, i.e., the efficiency has not declined [98]. Another novel method for enhancing electric conductivity by incorporating PEDOT:PSS with carbon paper at the interface of HTL and CE was reported by Teixeira et al. [99]. Although conventionally, most perovskite solar cells have been fabricated with Au and Ag-based CE, but due to their high price and complex processing, novel materials have gained attention. The few novel materials such as CNTs, graphene, CQDs, coal, graphite, organic, inorganic, and polymeric materials have been providing reasonable solar parameters compared to those of conventional materials for solar cells. To improve the efficiency of the solar system, various techniques have been incorporated, such as different coatings, different concentrations of materials, doping, composites, and variable temperatures for proper sealing. To reduce the fabrication cost, most of the researchers have used HTL-free PSC, where the cell’s same layer works both HTL and CE. Thus, the C-CEs have less efficiency, but they have several advantages over conventional materials, such as cost efficiency, long-term stability, and hydrophobicity.

## 3. Effect of Nanomaterials on the Performance of the Perovskite Solar Cells

Nanomaterials have been playing an indispensable role in various fields, such as water treatment, food packaging, biomedicine, electronics, agriculture, and energy generation [100,101,102,103,104,105,106,107]. Nowadays, these materials have been applied to all generations of solar cells, especially third-generation solar cells such as dye-sensitized solar cells, perovskite, and organic and inorganic cells [108]. These are widely accepted due to their cost-effectiveness, size, morphology, electrical and optical properties, and environmentally friendly nature [105]. However, these are incorporated in various components of solar such as semiconductors at the photoanode, ETLs, HTLs, and CEs. For example, the commercially available carbon paste and MWCNT were incorporated for HTL-free PSCs and compared in terms of the solar parameters. Where the Jsc and Voc of the carbon paste and MWCNT are 11.1 mA/cm^2^ and 13.77 mA/cm^2^, and 750 mV and 780 mV, respectively. The carbon paste may bother the stability in contrast to MWCNT due to its close interaction with perovskites [83]. A CsPbBr_3_ NPs in chlorobenzene solution incorporated in the modification of the light-absorbing layer of perovskite boosts the solar parameters. These NPs modify light absorber film (MAPbI_3_) into Cs_1−y_ MA_y_ PbI_3−x_ Br_x_ NPs. Although this may make the interface easier for transportation charge carriers, this passivation with the NPs may have many other possibilities, such as better crystallinity of perovskite, morphology, and layer quality as CsPbBr_3_ NPs lie at the nucleation center of the perovskite. The best efficacy of this cell was found to be 20.46% [109]. Carbon nanomaterials can have better compatibility with organic and inorganic materials. Therefore, they can enhance the charge carrier’s mobility. For example, a carbon nanotube (CNT) was inserted with CuSCN as the CE for inorganic PSC [89]. It has achieved high light endurance at 100 mW/cm^2^ over 1000 h and produces 17.58% efficiency. Therefore, due to the light endurance of this composite for long time intervals, it may reduce the radiative recombination loss [89].

Among nanomaterials, carbon nanomaterials are quite popular in energy generation systems. These materials have gained popularity in solar energy due to their cost-effectiveness, flexibility, ease of use, hydrophobicity, and high efficiency [83,104]. The examples of carbon nanomaterials widely applied in perovskite solar cells as CE are namely (i) MWCNT, (ii) carbon nanocoils (CNC), and (iii) graphene. These provided the highest efficiency, HTL-free PSC, decreased radiative recombination loss, and improved passivation. Recently, Gao et al. [58,96] have used nanomaterials such as carbon nanotubes (CNT), nanocarbons (NC), and graphene (GN) for C-CE. The overall solar parameters are tabulated in Table 3. The ethyl acetate-based anti-solvent coated carbon quantum dots (EACQDs) with variable concentrations (0.005, 0.01, 0.02, 0.05 mg/mL) were incorporated in surface passivating anti-solvent as well as CE [97]. The efficiency of the anti-solvent treated nanomaterials has improved by over 10% compared to the pristine nanomaterials constructed with PSC [97]. The solar parameters, such as Voc, Jsc, FF, and are tabulated in Table 3. The anti-solvent treatment of nanomaterials is an exceptional scheme in order to enrich the stability, shrink electron recombination at the interface, and improve solar performance. E.g., oxidized MWCNTs dipped into the anti-solvent have gained compatibility with perovskite and diffuse the electrons reaching out from the load. Application of this technique has delivered minimal degradation in terms of stability over 500 h [110]. Furthermore, the MWCNTs constructed HTL-free PSC provided 15.56% efficacy which was found stable in a humid environment and retained 80% of initial efficiency [20]. The performance of the solar cell not only increases with carbon nanomaterials. The composite or doping may have better possibilities. For example, Guo et al. [12] described the CE as a composite of CNT and CF (carbon fiber). The CNT-based electrode provided 8.93% efficiency, whereas the CNT/CF gave 11.80%. Additionally, the composite electrode has been found to be superior in terms of PCE and FF to the CNT alone. This composite has improved the hole carrier mobility and hole excitation at the interface of perovskite and CE. The nanomaterials play a vital role in ejecting the photon-generated electron to the back electrode in PSC. The nanomaterials have been used in different constituents of PSC. For instance, the SnO_2_ and core-shell ZnO-SnO_2_ are employed as ETL, where the core-shell NPs have better efficiency than single SnO_2_ NPs. The efficiencies of core-shell ZnO-SnO_2_ and pristine SnO_2_ as ETLs were 11.66% and 14.35%, respectively. The core-shell NPs surge other solar parameters than single NPs due to the perfect energy alignment with the active layer. As a result, they also have better stability and electron injection pathway [111]. Guo et al. [30] reported that nickel-doped graphene (Ni-G) is suitable for HTL. They have also testified other materials such as pristine graphene and carbon. It was found that the Ni-G-based HTL for inorganic C-PSC produced the best efficiency of 10.90%. The higher efficiency may be due to the compatibility of energy alignment between HTL and perovskite and HTL and CE, which enhanced the quick movements of electrons and holes at that interface. Similarly, Ramli et al. [13] employed graphene for the perovskite passivating layer at various concentrations (0.6, 0.8, 1.0, 5.0, and 10.0 mg/mL). Their study showed that employing graphene cannot provide adequate efficiency; however, it has retained 90% of its initial efficacy for 60 h. The carbon nanomaterials could also work as masking materials that focus the UV light onto the light-harvesting component of PSC. For example, the carbon quantum dots (CQDs) employed with poly (methyl methacrylate) (PMMA), i.e., CQDs-PMMA as masking material on top of the light-harvesting layer of a solar cell where the layers were spun coated at ambient temperature. When the cells with and without this masking material were compared in terms of efficiency, it was found that the cell with PMMA-CQDs provided 17.86% efficiency, whereas the cell without PMMA-CQDs contributed 16.4%. Overall, solar parameters for the masking material are higher than those of the non-masking material. Additionally, these PMMA-CQDs have low-conversion fluorescence properties and are responsible for better morphology, ease of fabrication, and cost-effectiveness. Due to the above enormous properties, these materials may have the possibility in future research and development [64]. Another novel fabrication included nanostructured perovskite architecture, which employed a thin layer of poly-dimethyl siloxane (PDMS) as a masking layer on top of a conductive glass substrate. This silicon-mediated texture layer on CE is called the fabrication condition considered structure (FCCS). The solar performance was compared with that of a conventional planar solar cell with a different layered structure of FCCS. It was found that the planar solar has Jsc 17.51 mAcm^−2^, Voc 960 mV, FF 82.42%, and η 13.86% whereas the FCCS has Jsc 22.45 mAcm^−2^, Voc 980 mV, FF 83.13%, and η 18.29%. The parameters of the proposed nanostructure solar cells were found to be significantly higher than those of the planar structure. The higher efficiency of the cell is due to PDMS materials’ ability to reduce the reflection of incident light on the substrate as it has a suitable reflective index. Although the matrix material is transparent, it provides more flexibility than a conventional solar cell due to its lower thickness [112]. The tungsten nanoparticles (WO_3_ NPs) embedded in carbon have been used as an interface in perovskite/CE. Two types of solar cells (with and without WO_3_ NPs fixed to carbon) were designed. The efficiencies of solar devices with and without tungsten NPs were 10.77% and 8.11%, respectively. This WO_3_ NPs-inserted structure was found to be significant for elevating the hole mobility in the device [113]. Copper nanoparticles (CuNPs) were testified for the cost effective alternative material of expansive Au CE [114]. Subair, et al. [115] has reported enhanced efficiency by doping of carbon nanodots (CNDs) in PC_61_BM. Similarly, for HTL free PSC, NiO NPs have been incorporated to carbon in order to boosts the efficiency [93]. However, the nanomaterials have been employed in the different structures of PSC, such as ETL, HTL, perovskite, and CE. The different structures of carbon and non-carbonaceous materials, including doping and composites, are considered the milestones for the energy generation materials for the PSC. Overall, these materials have delivered better solar parameters, stability, hydrophobicity, ease of fabrication, cost-effectiveness, and environmental friendliness.

## 4. Effect of Plasmon Nanoparticles on the Performance of Perovskite Solar Cells

The noble metal nanostructure can be considered a quality improvement material for energy conversion as it induces optical properties, electron separation, and better interfacial strength with the perovskite layer [18]. For instance, Deng et al. [116] prepared core-shell-mediated nanocuboids (Au-Ag/SiO_2_ core-shell NPs) and applied as ETL with various concentrations (0, 0.5, 1.0, and 1.5 vol%). Among different concentrations of Au-Ag/SnO_2_, 1.0 vol% showed the best performance. The efficiencies of a 1.0-volt-based solar cell and the control were 17.38% and 15.41%, respectively. Besides the efficiency, there is a dramatic improvement in the short circuit current density of core-shell metal nanocuboids compared to that of the control. The JSC for the Au-Ag/SiO_2_ core-shell NPs and control solar cells were 23.78 mAcm^−2^ and 20.78 mAcm^−2^, respectively [116]. The plasmonic effect of Au NPs has been tested. The solar cell incorporating Au NPs as an ETL significantly improved the Voc and Jsc compared to the cell containing pristine TiO_2_/TiO_2_ ETL. Additionally, the cell consisting of TiO_2_/Au NPs/TiO_2_ as ETL resulted in Voc 1070 mV, Jsc 22.3 mAcm^−2^, and 19.42%, while for TiO_2_/TiO_2_ ETL, Voc 1040 mV, Jsc 20.9 mAcm^−2^, and 17.76% were obtained [8]. The successive enhancement in performance by incorporating Au NPs in ETL can have the following strategies: (i) core-shell Au NPs having a monodispersing plasmonic effect result in reasonable shell wideness; (ii) in the precursor solution, incorporated star-shaped block co-polymers (nanoreactors) with precisely controlled sizes; and (iii) provided the quality design due to the favorable composition of the ETLs. This modelling could be one of the possible efficiency enhancement techniques for the future study and commercialization of perovskite solar cells [9]. In the perovskite solar cell, nanoparticles with different structures, such as nanorods, core-shell particles, spheres, nanotubes, etc., have been introduced, especially in the case of Plasmon NPs, who is sphere-shaped and core-shell-based structures are most often in use. The introduction of plasmonic nanostructure exclusively at an interface of PSC can have several advantages, such as: (i) inserted NPs have a high electrical field at the interface of PSC, which may enhance light absorption; (ii) reduce the interfacial recombination of charge carriers; (iii) perfectly passivate the layer as a consequence of energy alignment matching with light-absorbing and other layers; (iv) induce the rate of exposure of charge mobility [117]. The dimensions of NPs may have a significant impact on their absorbance of light. The nanoparticles with a diameter of 20–60 nm and without Au NPs were tested for the PSC. It was found that the absorbance of the cell at 60 nm had the highest absorbance of all the diameters tested, with or without plasmonic NPs. However, 20% absorbance was improved for solar cells with Au NPs of diameter 60 nm [117]. The plasmonic Au NPs were incorporated in ETL, perovskite, and HTL; among these three layers, Au NPs best fit with the HTL as it has produced an efficiency of 15.80%, which is higher than ETL and perovskite. Even the other solar parameters were found to be higher for Au NPs that incorporated HTL. Additionally, the Au NPs with variable diameter (30, 50, and 70 nm) were placed in a perovskite layer and the best absorbance was found to be at 30 nm [118]. Besides the absorbance, the solar cell with a diameter of 30 nm has produced the best efficiency (16.20%) among these three diameters. Therefore, the plasmonic Au NPs can have a significant role in the absorbance of light by generating an electrical field on their own surface [118].

The concentration of nanomaterials during preparation plays a key role in the performance of the PSC [119]. The core-shell nanostructure of Au-Pt-Au with various concentrations (0, 0.5, 1, and 2 wt%) has been incorporated in ETL [120]. It was found that 1% of concentration (by weight) had significantly better efficiency than the other concentrations. The increased concentrations in the cell of more than 1% were found to decrease due to the interfacial recombination of electron-hole pairs [120]. Another core-bishell nanostructure (Ag-TiO_2_-fullurene) was found efficient for inorganic PSC [119]. It produced almost 20% more efficiency than without a core-bishell structure. Core-shell has played a substantial role in improved efficiency. As Ag NPs induced the light absorption at the perovskite, the TiO_2_-fullerene bi-shell made a channel for charge carriers to reach the substrate with the help of fullerene. Furthermore, by employing these core-shell NPs in the light-harvesting layer and tuning the plasmonic coupling effect, the efficiency shifted from 13.0% to 20.2% [119,121]. However, the plasmonic NPs incorporation into the different layers of perovskite could be the breakthrough for stable and efficient PSC. It mainly heightens the light absorbance capacity of perovskite and provides better shape, size, morphology, and energy alignment.

## 5. Characterization of Perovskite Solar Cells

There are several tools and techniques to characterize the optical and electrical properties, structure, size of the particles, and morphology of PSC. Such techniques are UV-Visible spectrometry, photoluminescence spectra (PLS), XRD, SEM, FE-SEM, EIS, etc., and solar cell characterization can be obtained with the help of the Solar Simulator (Keithley series). Here, a few of the characterization techniques for PSC are mentioned below.

### 5.1. UV-Visible Spectroscopy/Photoluminescence Spectra in Terms of Efficiency

**Figure 2:**
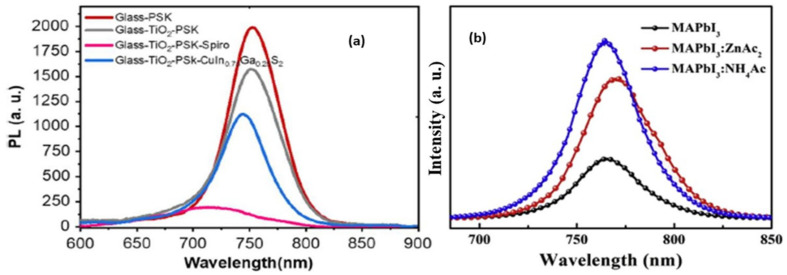
Ref. [101] PL spectra of (**a**) the light absorption was observed when inserting ETL but no absorption after injecting Spiro-OMeTAD as HTL as the expected result. When CuIn0.75Ga0.25S_2_ was injected as HTL, the high intensity was observed so that the light goes beyond perovskite to generate electrons, and holes may disturb the function of HTL [101]. Reprinted with permission from Solar RRL © 2020 WILEY–VCH Verlag GmbH & Co. KGaA, Weinheim, Germany. (**b**) PL spectra of pristine perovskite and two precursor solvents, ZnAc_2_ and NH_4_Ac, were added to the perovskite. This illustrates that the higher the perovskite intensity, the better the efficiency [65]. Reprinted with permission from the Journal of Alloys and Compounds © 2019 Elsevier B.V.

It is considered that the better the light absorbance ability of perovskite, the higher the performance of the solar cells. Although the stability of the PSC is considered one of the key factors, after exposure to light, its efficiency declines in most cases [2,23]. The four different TiO_2_-based ETLs have been prepared with the variable concentration doping of SiO_x_, i.e., TiO_2_/Si_x_ (where x = 0, 0.5, 1.0, and 2.0%) [81]. The cells were tested at 65 mW cm^−2^ for 30 min. It was found that 1% (by weight) of coated ELT provided the best efficiency compared to the other concentrations [68,81]. The light absorbance of perovskite depends on the nature of ETLs. For instance, two types of ETLs were incorporated for PSC [51], where the first ETL contains pure SnO_2_ and the second contains a SnO_2_-KCl composite. The UV-visible absorbance studies revealed that the absorbance of composite ETL is greater than that of pure ETL. Though in terms of efficiency, the cells containing the SnO_2_-KCl composite were found to be superior to pure SnO_2_. Therefore, it could be confirmed that a high absorbance of semiconducting NPs used in an ETL results in greater solar parameters [51]. Photoluminescence (PL) studies the interaction of light with particles at different wavelengths (200–1000 nm). Li et al. [97] reported the EACQDs in the solution formed with pure and three other concentrations (0.005, 0.01, 0.02, and 0.05 mg/mL) inserted as perovskite surface passivation. The concentration of 0.01 mg/mL was found to be better than other concentrations due to its higher absorbance. Then PLS and UV-visible were carried out for 0.01 EACQDs and pristine solar cells. It was found that 0.01 EACQDs-incorporated PSC passivation displayed a higher absorbance in the entire visible light region than that of pristine PSCs, thereby resulting in high efficiency. HTL-free PSC-containing carbon materials such as carbon nitride (CN), Acetylene Black (AB), nanocarbon (NC), and graphene (GN) were tested as bridging materials for the PLS study [96]. The PLS of these carbon materials provided the intense peak at 770 to 780 nm, where the highest intensity peak was found for the CN. The high absorbance clearly shows that increasing charge injection has the consequence of reducing radiative recombination and increasing charge transporter generation. However, carbon perovskite solar with HTL-free CN CE provided the best efficiency, i.e., 15.09% [96]. Steady-state PL can also study the interfacial optical properties of materials such as perovskite/ETL and perovskite/HTL with and without the accumulation of an additive layer. When the MAPbI_3_ perovskite was tested with two additives such as NH_4_Ac and ZnAc_2_, the PL intensity of the solar cell consisting of NH_4_Ac was found to be significantly higher than that of ZnAc_2_ [65]. This may be due to the following properties of NH_4_Ac: (i) reduced defect-induced recombination; (ii) improved crystallinity; and (iii) the redshift of the intense peak of this material results in a difference in bandgap energy. The higher the PL peak, the higher the efficiency, i.e., MAPbI_3_:ZnAc_2_ and MAPbI_3_:NH_4_Ac showed 13.88% and 12.30% efficiency, respectively [65]. A UV-vis absorption study revealed that the ZnAc_2_ employed layer provides an entirely higher absorbance than NH_4_Ac [65]. This is due to the fact that the Zn ions may enhance the light absorption capability of the ammonium ions. However, due to the big void in its structure, it has a poor morphology compared to that of NH_4_Ac, which reduces its efficiency [65]. Lv et al. [33] reported that the pre-heating temperature of the deposition film may affect the performance of perovskite solar cells. The UV-visible absorbance of the ELT (m-TiO_2_) with different preheating temperatures (293, 313, 333, 353, and 373 K) was tested. It was found that the best absorbance for the film was until 333 K, after which it was found to decrease until 373 K. The decrease in absorbance may be due to the inadequate crystallinity of ETL materials. To apply CuSCN as HTL, the interactions between perovskite and CuSCN were analyzed with the help of PL spectra [33], and the intense peak was found at 769 nm. Thus, the intensity of the cell incorporating CuSCN was found to be decreased due to the deposition of HTL into perovskite, which induced hole collection and consequently resulted in higher efficiency than without CuSCN [33]. The PL of different modification of HTL in Figure 2a and passivation of perovskite with two solvents are illustrated in Figure 2b respectively. 

The optical properties of the CEs incorporated with various concentrations (0, 0.5, and 1.0 mg/mL) of O-MWCNTs [109]. Among these concentrations, 0.5 mg/mL provided the best absorbance at 400–550 nm. On the other hand, the UV-visible absorbance of O-MWCNT showed a similar absorbance pattern to that of perovskite at about 780 nm. Thus, the higher the optical absorbance, the better the efficiency, as well as this CE provides better JSC than other concentrations and a pristine structure [110]. The steady-state PL study of 0.5 mg/mL O-MWCNT shows the highest intensity in the 743 nm wavelength range for the light-absorbing layer. The enormously increased intensity at this concentration may be beneficial for reducing the charge trapping density and conquering the charge non-radiative recombination loss at perovskite [110]. However, the different attempts, such as increasing the concentration of the deposited solution, increasing the annealing temperature of the ETL, creating a composite structure, treating the perovskite with variable anti-solvents, and inserting the layer with variable thickness, enhancing the PLS and UV-visible absorbance.

### 5.2. Electrochemical Impedance Spectroscopy (EIS) of Perovskite Solar Cells in Terms of Efficiency

**Figure 3:**
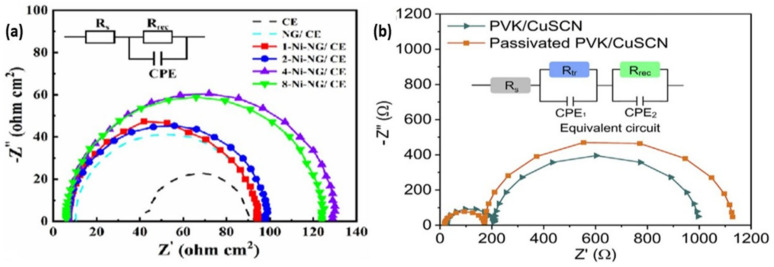
Nyquist plots of PSCs. (**a**) Pristine CE, NG/CE, and variable concentrations (1, 2, 4, and 8 mg/mL) of Ni-doped in NG (Ni-NG). Bigger semi-circle present in high-frequency regions represent higher R_ce_ values, and the concentration of Ni-NG at 4 mg/mL provided the biggest semi-circle followed by 8 mg/mL, resulting in the highest efficiency according to [30]. (**b**) It illustrates the conductivity of perovskite/HTL and passivated perovskite/HTL. The small semi-circle is at a high-frequency region, while the larger semi-circle is at a low-frequency region. Both have similar R_s_, lesser R_tr_ and higher R_rec_. As bigger semi-circles, the passivated perovskite/CuSCN has a faster rate of charge transfer and minimum energy loss than the control [31]. Reprinted with permission from Materials Today Energy © 2021 Elsevier Ltd.

EIS is an important characterization technique that measures or estimates the charge transport in an entire cell as well as the charge transportation through different interfaces such as ELT/perovskite, perovskite/HTL, HTL/CE, and FTO to CE. EIS may also be helpful in determining the series resistance (R_s_) and shunt resistance (R_sh_) status at the different interfaces of the perovskite solar [122,123]. For instance, the different concentrations (0, 0.5, and 1.0 mL/mg) of O-MWCNTs were incorporated in CE in the initial condition the device was illuminated under (bias voltage 0.3 V under AM 1.5 @ dark). In the real situation, in an initial case where the R_s_ was increased, the formation of a semicircle relates to the upsurge of transfer resistance (R_tr_), which is related to the charge transmission at an interface between perovskite and CE. At the same condition but different concentrations of O-MWCNTs, there were small R_s_ and R_tr_. When these electrodes were treated with an anti-solvent, R_s_ and R_tr_ dramatically increased from 800 ohms to about 3500 ohms. Furthermore, the arc in Nyquist plots provides information about the interaction between different interfaces of perovskite through interfacial recombination resistance (R_rec_). In this regard, the cell consisting of 0.5 mg/mL O-MWCNTs provided a high R_rec_ and a low R_tr_. Thus, the perovskite solar cell with higher R_rec_ and lower R_tr_ indicates the enhanced interaction between perovskite and CE [110]. Similarly, Guo et al. [21] have reported that the EIS for the different CEs, such as CF, CNTs, and CF/CNTs, was performed at a high-frequency range because the low frequency (10 Hz) caused several interfacial recombinations. For this measurement of the accepted frequency (10 Hz–1 MHz under the dark at 0.6 V forward bias), the three Nyquist plots were obtained with two semicircles in each. The obtained semicircles are analyzed based on the different frequency ranges: (i) the semicircle obtained in the high-frequency range (106 Hz–104 Hz) symbolizes the charge recombination (R_rce_) at perovskite/CE; (ii) the second sort of semicircles obtained in the lower frequency range (104 Hz–10 Hz) symbolizes charge recombination at TiO_2_/perovskite [99,113]; and (iii) the high-frequency (1 MHz) divert to the actual axis that symbolizes series resistance R_s_. It is clearly seen that the dissimilar CE produces a lower number of R_s_, which could result in better FF and efficiency because it boosts conductivity. Furthermore, the lower the R_rce_ value, the better charge transport in the Perovskite/CE interface [12]. In another study, the EIS was conducted for three common sorts of C-CEs, such as vulcanized carbon (VC), mesocarbon (MC), and superphosphate (SP), under the constant illumination of light at 0.6 V. The R_s_ of the VC and MC are almost similar, while the SP R_s_ are slightly higher, which corresponds to the reduction of PCE. In the case of R_rce_, the value of SP is extremely higher than the other two CE [124]; therefore, a higher value of R_rce_ also results in lesser efficacy, as shown in Table 4. The Nyquist plots of PSCs with variable modification of CE in Figure 3(a) and with and without passivation of perovskite in Figure 3(b) respectively. 

The EIS was conducted for the solar cell containing FTO, CH_3_NH_3_PbI_3_, Spiro-OMeTAD, graphene, and Ag interfaces [125]. Two cells with the same structure but a modification in HTL (layer passivation) were carried out, one with graphene added in between HTL and CE and another without graphene. The high-frequency range with the first semicircle arc allotted electron transport resistance (R_tr_) at the interface of perovskite to CE, and the low frequency with the second semicircle range signified recombination resistance (R_rce_) at ETL/perovskite and FTO/ETL. It was found that the addition of graphene has enhanced the impedance, and the R_tr_ value mainly depends on the thickness of the graphene. The thicker graphene layer coating reduces the R_tr_; therefore, it reduces the hole mobility in the solar cell, the Jsc, and the efficiency of the PSC. Regardless of the coating of CE or passivation in different layers, the composite structure may have a significant role in the performance of PSC. The composite electrode and doping electrodes demonstrate comparatively less R_s_ and R_rec,_ which are responsible for improving charge mobility, declining charge recombination, and consequently enhancing Jsc and FF [30]. Chu et al. [126] reported that high R_tr_ affects the improvement efficiency of the solar cell by reducing the rate of charge recombination at the ETL/perovskite interface. A study on the doping of NiO:C in different ratios (1:10, 1:20, and 1:30) was reported by Chu et al. [126]. It was found that the best matching ratio for doping was 1:20 based on Nyquist plots. The frequency ranges are described as follows: (i) the high-frequency range revealed that the charge transportation at Perovskite/CE; (ii) the low-frequency range revealed charge transportation at ETL and perovskite. When NiO:C is doped in 1:20, it depicts the formation of the shortest semicircle (high-frequency range), which specifies a good ratio of charge transport as well as hole transportation, resulting in advanced Jsc and FF of PSC [93]. The effect of a water-treated carbon electrode-based cell in comparison to the pristine cell was observed by EIS [127]. The high-frequency region of the semicircle demonstrated the R_tr_ at TiO_2_/perovskite, which is not affected by the treatment of water. In contrast, at the low-frequency region of the semicircle, water treatment encourages charge recombination and decreases the R_rec_ at ETL/perovskite. Conversely, a water-treated cell has provided the best efficiency [127]. The smaller value of R_rec_ gives the best efficiency [81]. The lower value of series resistance (R_s_) and higher shunt resistance (R_sh_) provided the best solar cell parameters [58]. The EIS study revealed the status of different cell interfaces in different ratios and concentrations. From this study, it is clear that the lower the values of R_s_ and R_ce_ and the higher the values of R_tr_ and R_sh,_ the better the mobility of electrons and the holes in all the interfaces of PSC that result in the best efficiency. The comparative values of R_s_ and R_rce_ in terms of the four solar parameters are illustrated in Table 4.

EIS is not only applicable for determining the status of charge transportation throughout the different interfaces of PSC. It is also helpful to determine the effects of light and heat and their responses throughout the interface. For example, the charge recombination in the light-absorbing layer or charge-transporting layer is a major weakness of the PSC, which may be resolved by an EIS study. For example, the central recombination site of the PSC was recognized by illumination-side-dependent impedance spectroscopy (ISD-IS) [27]. In this study, the device was illuminated from ETL and HTL on both sides, and the charge recombination resistance (Rrec) was calculated. At high current and voltage, the recombination at the ETL side was found to be higher than that at the HTL side. This technique is beneficial for preparing recombination-free, stable, and efficient solar cells [126].

### 5.3. X-ray Diffraction Analysis (XRD) of PSC in Terms of Efficiency

**Figure 4:**
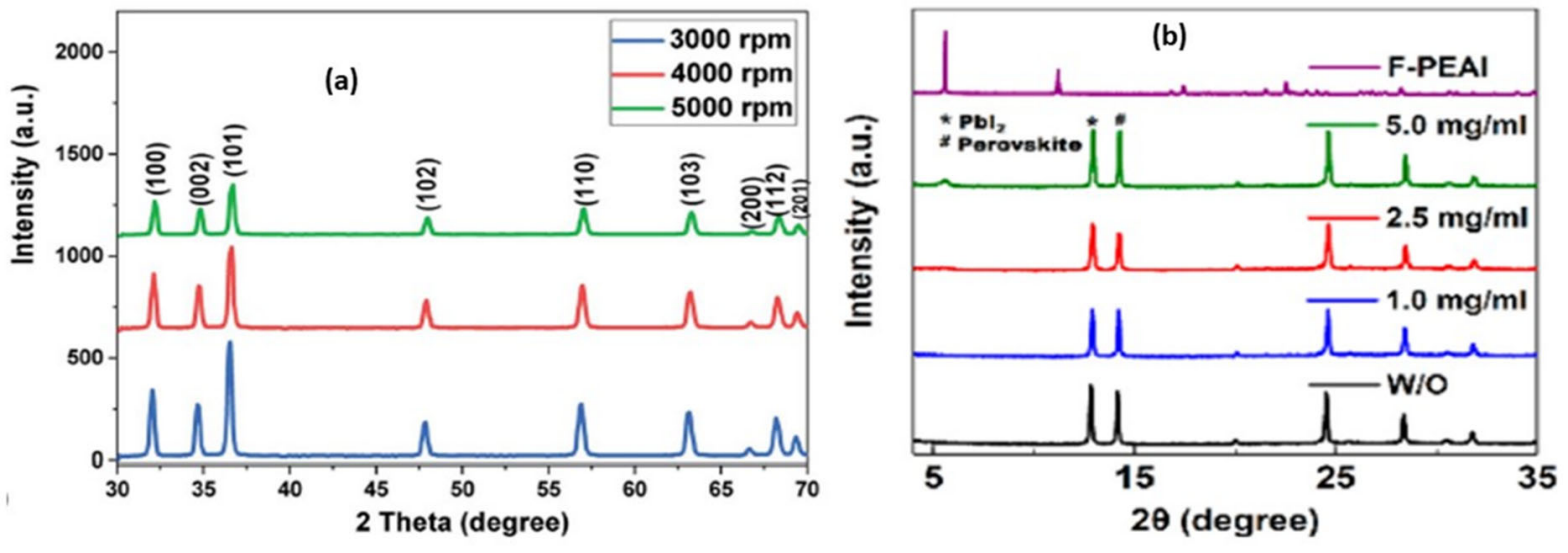
XRD images of (**a**) the spin coating rotation speed 3000, 4000, and 5000 RPM of ZnO on a substrate. The diffraction angles were found to be decreased when the rotation increased. This illustrates that in high-speed depositing solution may eliminate from the surface of the substrate due to the small area simultaneously reducing the thickness of the layer as well the crystal packing [20]; (**b**) inserting precursor solution (F-PEAI of concentration 1.0, 2.5, and 5.0 mg/mL) in between perovskite/HTL and without F-PEAI. As the concentration of F-PEAI increases, the crystallinity also increases, indicating the highest peak [68]. Reprinted with permission from Applied Materials Copyright © 2021, American Chemical Society.

The XRD studies the crystallinity of the materials used in perovskite, whether they are ETL, HTL, or passivating materials. Generally, higher crystallinity of the perovskite and better surface passivation results in better light absorption, reduced surface charge recombination, and increased electron-hole mobility [22]. For instance, by incorporating PEDOT:PSS in ETL and nitrogen-doped CQDs (N-CQDs) in HTL, it can be scrutinized the effect of both materials’ crystallinity on the light-absorbing layer (MAPbI_3_) [78]. It was seen that the dissimilar concentrations of N-CQDs (1, 3, and 5%) deliver a significant improvement in crystallinity as the volume of doping (N-CQDs) increases and peak intensity is found to increase. Although the solar cell at 3% N-CQDs doped with perovskite had the best compatibility that matched the crystallinity of PEDOT:PSS, it had the highest efficiency [78]. The solar cells fabricated with and without SWCNTs as CEs were analyzed for MAPbI_3_ light-absorbing material [34]. It was observed that the three major intense peaks at 14.48°, 28.39°, and 32.03° specified the crystalline structure MAPbI_3_. By incorporating SWCNTs, crystal growth is not distressed [34]. Similar CQDs with different concentrations (0.1, 0.15, and 0.2 mg/mL) of precursor solutions have been used in perovskite’s active layer [127]. The most efficient cell was found at 0.15 mg/mL of CQDs, and the crystal structure of this was compared with the pristine cell’s crystal structure after seven days at 85% humidity by XRD. After a week-long continuous exposure to light, the pristine cell was degraded and retained 20% of its initial stability, while the cell consisting of 0.15 mg/mL CQDs retained over 80% of its initial stability. Thus, it is clear that inserting the precursor solution improved the perovskite film quality and acted as a hydrophobic layer against moisture, as it has shown the same intense peak as the initial. It acts as a hydrophobic layer due to the presence of functional groups such as −CH_2_−, C = C, and >C = O, so this passivation is considered the best grain boundary passivation [128]. The XRD of different speed of spin coating and comparative XRD analysis of variable concentration of precursor solution are shown in Figure 4.

Two electrodes consisting of PbBr_2_ serve as the light-harvesting layer [70]. Both are dipped into the precursor solution consisting of CsBr/Methanol in two different ways: (i) face-up dipping and (ii) face-down dipping. The immersion of electrodes into the precursor solution mainly depends on the immersion period. In the case of face-up immersion, the formation of CsPbBr_3_ is faster (around 5–6 min) when immersion of PbBr_2_ into CsBr/methanol occurs, but the molecules simultaneously fall apart. Therefore, the diffraction peaks related to the CsPbBr_3_ layer disappear, and then apparently, CsBr diffraction peaks formed [70]. Similarly, when the electrode is immersed into the precursor solution facing downward, it basically takes more time to deposit (the dipping period is 10, 20, 30, 40, 50, and 60 min). The film formation rate is increased until 40 min (grain size increases) and then slightly decreases. The largest grain size at 40 min was found to be about 850 nm, whereas, at 60 min, it was almost 500 nm. The grain size formed at 60 min was slightly bigger than at 10 min (average grain size vs. dipping time plot observation). However, in face-down immersion, there is less chance for the film to disintegrate as well as less probability for the re-formation of CsBr diffraction patterns [85]. Another technique that provides the best crystalline structure for PSC is top-seeded solution growth (TSSG). Based on this technique, thmine perovskite layer (CsPbI_2_Br) was deposited by spin coating and then annealed at different temperatures (0, 150, 200, and 250 °C) [35]. Herein, the TSSG-based annealing of perovskite gains crystallinity as temperature increases (150, 200, and 250 °C), and the initial diffraction peaks at 100 and 200 shift towards higher angles of diffraction. This may be due to two reasons: (i) compressive strain enlargement of TSSG CsPbI_2_Br in the horizontal direction, and (ii) the halide interchange reaction adopted Br^−^ anions to the bulk structure [35]. The XRD analysis of the PSC with CEs (low-cost carbon CE) at variable annealing temperatures (100, 200, 250, 300, and 400 °C) is observed [129]. By increasing the annealing temperature, the diffraction angles also increased, but the interlayer spacing between the grain boundaries decreased. It also clearly shows that the reduction of interlayer spacing results in better crystal growth formation, i.e., a proper gap filled by the carbon at the interface of perovskite and CE. The highest efficiency obtained from the cell containing CE annealed at 400 °C is 13.3% [129]. When the concentration of perovskite precursor increased, crystallinity and grain size were also found to be increased [130]. The diffraction angles were found to increase when methylammonium chloride (MACl) precursors with various concentrations (0.15, 0.3, 0.45, 0.6, 0.75, 0.9, and 1.05 mmol) were added to 1 mL of MAPbI_2_. The concentration of MACl increased up to 0.45 mmol, and then the crystallinity was found to increase, whereas, after that, the crystallinity decreased to 1.05 mmol. Therefore, the highest efficiency of the PSC was found at 0.45 mmol, i.e., 14.50%, which is double that of the control cell. It is clear from this study that a limited concentration range of precursor solution could only provide better crystallinity of the perovskite as well as PCE [131]. For better PSC crystal structure formation, the electrode immersion techniques, suitable concentrations of precursor materials, annealing temperature, binding group present in the passivating material, and speed of deposition of the precursor solution on an active layer are important. Thus, the better the crystal structure of layered materials, the better the efficiency of PSC.

### 5.4. Morphological Study of Perovskite Crystals

The morphological study is mainly carried out by high-resolution microscopes such as atomic force microscopy (AFM), scanning electron microscopy (SEM), transmission electron microscopy (TEM), field-emission scanning electron microscopy (FESEM), etc. It provides a clear picture of the crystal growth in a substrate, the formation of layers, and the thickness of the layer. For instance, the top-seeded solution growth (TSSG) technique for CsPbI_2_Br was carried out under various conditions (control, 150, 200, and 250 °C) [35]. The SEM and AFM images show clear crystal growth when the electrodes are heated over 150 °C. The grain passivation boundary has no gaps, as grains are attached smoothly [35]. The porous graphitic carbon (GC) was extracted from an invasive plant called Eichhornia crassipes (EC), and then three different CE-GC counter electrodes were prepared for HTL-free CE [132]. These cells were annealed at three different temperatures (450 °C, 850 °C, and 1000 °C), and then their morphological analysis was carried out by FESEM. Based on the sintering temperature provided on EC-GC, the dissimilar morphology is shown as follows: (i) At 450 °C, it displays the development of bulky and accumulated graphitic (ii) at 850 °C, it shows the fibrous configuration, pasted porosity, and declining particle dimension; (iii) at 1000 °C, the fibrous configuration alters into a skinny film of EC-GE. The variation in the configuration at 450 °C is due to the occurrence of partially reacted residues of EC-GC; at 850 °C it may be due to the residual content of EC-GC evaporation, and at 1000 °C it may be due to the modification of crystals in a thin layer due to the high temperature. Thus, incorporating a higher temperature is beneficial for enhancing the morphology of the carbon graphite, but it also helps to remove the unwanted impurities of EC-GC and stabilize the PSC. Overall, cells are found to be efficient, with the highest efficiency for cells annealed at 1000 °C [112,133].

**Figure 5:**
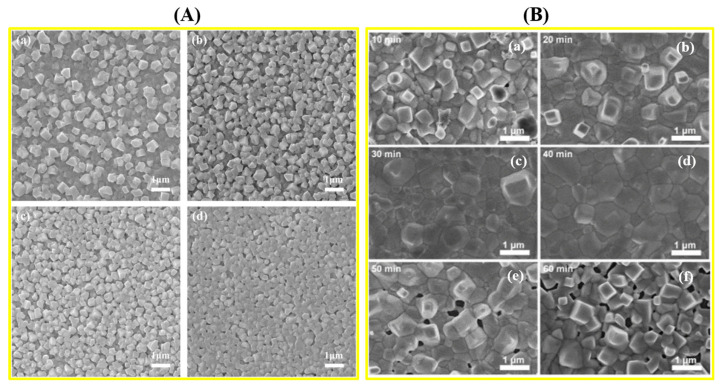
(**A**) Scanning electron microscopic (SEM) image of CH_3_NH_3_PbI_3_ active layer (a) without Au-Ag-SnO_2_ core-shell nanocuboids (CSNCs), and with (b) 0.5% (c) 1.0% (d) 1.5% additives of Au-Ag-SnO_2_ CSNC. Without the addition of CSNCs, high surface roughness while adding slightly the roughness found to be removed and increase the compatibility with perovskite [116]. Reprinted with permission from Journal of Power Sources © 2019 Elsevier B.V. (**B**) The SEM images of PbBr_2_ coated electrode dipped into a precursor solution of CsBr/methanol at 50 °C for 10, 20, 30, 40, 50, and 60 min image (a–f). Increasing the immersion time from 10 min to 20 min, smaller sizes of grains appear. When the immersion time increases over 20 min to below 50 min, bigger grains are formed and decrease the roughness between the crystal. The pinholes appeared when the immersion period increased over 50 min [85]. Reprinted with permission from Applied Materials Copyright © 2018, American Chemical Society.

FESEM was applied to figure out the average thickness of TiO_2_ as well as Al_2_O_3_ in the PSC with FTO/c-TiO_2_/m-TiO_2_/m-Al_2_O_3_/CH_3_NH_3_PbI_3_/WO_3_/carbon-based architecture [113]. The thickness of these ETL (TiO_2_ and Al_2_O_3_) layers is about 700 nm and 500 nm, respectively [78]. In another study, graphdiyne (GD) was incorporated as a host material in between the perovskite with various molar ratios (1:1:0.1, 1:1:0.25, and 1:1:0.4) of PbI_2_: MAI: GD [14]. The SEM images of such perovskite showed that the controlled morphology of the crystal structure had some pinholes and gaps in the grain boundaries while employing the GD improved the morphology. In the obtained morphology, the boundary gap has been filled up by increasing the ratio of GD, and the best morphology is at 1:1:0.25, then a few pinholes are observed when the concentration reaches 1:1:0.4. At 1:1:0.25 concentrations, the cell has provided the highest efficiency, i.e., 21.01% [115,116,117]. The different concentration of additives and without additives and effect of temperature in perovskite precursor solution are illustrated in Figure 5A and Figure 5B respectively.

The PSC was fabricated with and without inserting a layer of F-PEAI in between the perovskite and spiro-OMeTAD, and its morphological study was conducted by AFM and SEM [68]. It was found that F-PEAI-incorporated structures have a smaller gap between grains than those without F-PEAI. The morphological roughness in between the gains is decreased by around 23.7–21.5 nm by F-PEAI, while without F-PEAI, it was 24.7 nm [68]. Similarly, two sets of PSC, one containing PEAI in the perovskite layer as a precursor solution and another without PEAI, were observed and compared by SEM [134]. It is clearly seen that the cell without PEAI has pinholes in between the crystal, so these pinholes may distress the charge mobility and induce radiative recombination. In the case of PEAI-incorporated perovskite, the smooth formation of crystals was observed, i.e., packing crystals together. This may provide enhanced electron and hole mobility, so the efficiencies for cells without PEAI and with PEAI are 6.34% and 10.20%, respectively [134].

Perovskite surface passivation is considered a key aspect of enhancing light absorption. The PSCs were prepared with and without using tris(pentafluorophenyl)boron (TPFPB) [72]. The morphology of these cells was analyzed by SEM. TPFPB, as a passivating material, had provided a tight crystalline structure, while without TPFPB, few voids were observed. Thus, the passivating material’s incorporated structure enhances electron and hole mobility, resulting in higher efficiency than a non-passivated cell [72]. The ETL deposition technique and coating speed on a substrate can play a significant role in crystal growth. For a device, SnO_2_ as ETL was coated on a substrate by spin coating with different rotation speeds of 3000, 4000, and 4000 rpm [20]. The morphology was analyzed by FESEM. It was found that the speed of coating increased from 3000 rpm to 5000 rpm, and the initially formed voids in the crystal lattice have been found to be healed. Hence, it was clearly seen that the higher ETL coating speeds (5000 rpm) resulted in better morphology of the crystals with the 12.81% efficiency of PSC [20]. Several factors in the morphology may affect the performance of PSC, such as the nature of the materials, the sintering temperature of ETL, perovskite, HTL, and CE, and the deposition speed, method, and passivation ratio of the precursor solution. Suitable temperature, coating techniques, material concentrations, and materials selection are mandatory to overcome such barriers.

### 5.5. Current-Voltage (J-V) Characteristics of Perovskite Solar Cells

Current voltage is required to characterize solar cells, which determines their strength and weakness with the help of their Voc, Jsc, FF, and efficiency. Each and every solar system parameter is crucial for increasing efficiency. The solar simulator is a device that characterizes solar cell illumination under 1 sun intensity or 100 mW cm^−2^ [99]. Two HTLs, such as poly[bis(4-phenyl) (2,4,6-trimethylphenyl)amine] (PTAA) and self-assembled monolayer (SAM), were investigated in a solar cell with MAPbI_3_ as the active layer (0.105 and 1.008 cm^2^) [135]. The effectiveness of PTAA was reduced from 17.33 to 11.92% when the active area was raised from 0.105 to 1.008 cm^2^. Similarly, the SAM’s efficiency decreased from 18.47 to 14.64% when its active area increased from 0.105 to 1.008 cm^2^. It might be due to the fact that the smaller areas have superior interfacial interactions between perovskite and other layers, and solar cells have negligible hysteresis in both the reverse and forward scanning directions [135]. The current and voltage of a solar cell have been highly influenced by the doping and composition of perovskite [38,56,73]. For instance, two dopants were evaluated on MAPbI_3_ perovskite, including potassium iodide (KI) at concentrations of 0 and 5% and ethyl ammonium bromide (EABr) at concentrations of 0, 10, 20, 30, 40, and 50% [136]. In comparison to other concentrations, it was discovered that 5% of KI doping with 30% EABr had the best efficiency (12.88%). Moreover, these two KI and EABr concentrations have produced better values of Voc and Jsc, which are 21.0 mAcm^−2^ and 942 mV, respectively [136]. Other materials, including ETL, HTL, and CE modification or passivation, could also improve the solar cell’s performance in addition to the active layer modification [52,76,137]. The table shows the many materials that have been used in the ETL, HTL, and CE, with variations in the Voc, Jsc, FF, and Efficiency.

**Figure 6:**
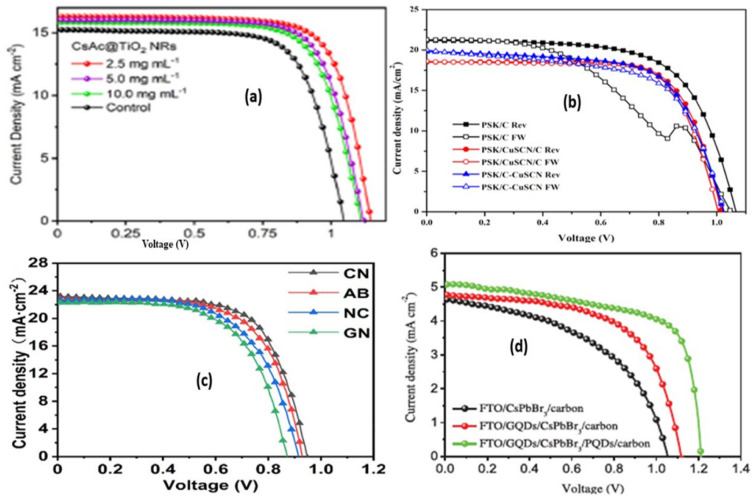
J-V characteristic curve of (**a**) modification of ETL by different concentrations of CsAc (2.5, 5.0, and 10.0 mg/mL with TiO_2_) and control. A suitable concentration is required to obtain the best efficiency [5]. Reprinted with permission from ACS Applied Energy Materials, Copyright © 2021, American Chemical Society. (**b**) Fabrication of PSC with various combinations such as Perovskite/C, Perovskite/CuSCN/C, and perovskite/C-CuSCN. In scanned forward and reverse directions, the highest efficiency was obtained for the cell incorporated perovskite/C combination in the reverse direction as the biggest J-V curve in figure [6] Copyright © 2019, American Chemical Society. (**c**) different carbonaceous bridging materials used as HTL-free PSC. The bigger the curve’s surface area, the higher the solar parameters are higher. The highest efficacy to lower based on the curve is CN > AB > NC > GN [96] Reprinted with permission from Journal of Colloid and Interface Science, © 2020 Elsevier Inc. (**d**) modification of solar in ETL and HTL with carbon CE [138]. Reprinted with permission from Small, © 2018 WILEY–VCH Verlag GmbH & Co. KGaA, Weinheim.

To improve the J-V properties, numerous unique passivating materials have been optimized in PSC [12,56,78,131]. The best performance for the PSC is provided by a few passivating materials, including polyaniline (PANI), tris(pentafluorophenyl)boron (TPFPB), ferrocenyl-bis-thiophene-2-carboxylate (FcTc_2_), 1-butyl-3-methylimidazolium tetrafluoroborate (BMIMBF_4_), and methylammonium succinate (MS) [137]. The variable carbon materials have been utilized as CE; most are used as carbon electrodes free of HTL, and a few are employed as passivates at the interface between CE and HTL [13]. For instance, perovskite is coated with solutions of various concentrations of graphene (0.6, 0.8, 1.0, 5.0, and 10.0 mg/mL), and the best concentration of the cell was compared to the control (Ag CE) [13]. Different graphene concentrations were shown to be less effective than the control. The J-V parameters of modification of ETL, perovskite, and HTL are illustrated in Table 5. The J-V plots for variable conditions applied in PSC ETL, HTL, perovskite, and CE are illustrated in Figure 6.

There are several materials that perform better than Ag-based CE [12,92]. The control is contrasted with the EACQD concentrations (0.005 mg/mL, 0.01 mg/mL, 0.02 mg/mL, and 0.05 mg/mL). All EACQD concentrations were discovered to be more effective than the control. The most effective cell has an efficiency of 15.14%, compared to 12.15% for the control, and contains 0.01 mg/mL of EACQDs. Concentrated EACQDs were shown to have greater values for all other metrics (Jsc, Voc, and FF) than pure solar [97]. Overall, new materials, including PANI, PTAA, PSM, TPFPB, FcTc_2_, BMIMBF_4_, and MS, are thought to improve all solar metrics. A few doping, composite, and modification techniques through incorporating and optimising an alternate material have been demonstrated for the successful development of PSC. The optimum circumstances for enhancing the effectiveness of the PSC include parameters such as temperature effects, material solvent treatments, and passivation. In terms of efficiency, carbonaceous materials are not ideal for manufacturing the majority of solar cells. However, because of their stability in the presence of moisture, air, and precipitation, these materials should be considered for future PSC research and development.

## 6. Energy Alignment of Different Constituents of PSC

Energy alignment in a solar cell is a crucial step for better electron and hole flow frequency and direction in the various PSC interfaces. Determining the HOMO and LUMO states is also advantageous. Each of the PSC’s constituents (FTO, ETL, Perovskite, HTL, and CE) has a different amount of energy. The perovskite serves as the focal point, donating electrons to one side while taking up holes on the other. The modified elements in PSC have varying energy levels, so altering each component with different materials could result in varying energy levels. Nonetheless, depending on their nature and type, those modifiers may either increase or decrease the band gap [62]. The energy orientation investigation of three distinct HTLs, including graphene oxide (GO), PEDOT:PSS, and polyaniline (PANI), was published in 2019 by Mabrouk et al. [144]. The position of HOMO energy is in the order of perovskite/GO/PANI/PEDOT:PSS/CE and −5.5 eV/−5.3 eV/−5.1 eV/−5.0 eV/−4.7 eV. All of these HTLs’ energies were revealed to match perovskite. Corresponding to this, the positions of the LUMO energies in the order of perovskite/GO/PANI/PEDOT:PSS/CE are respectively −3.95 eV/−3.5 eV/−3.05 eV/−3.5 eV. However, these three materials have an increasing order of energy in the following sequence: GO < PANI < PEDOT:PSS. The HOMO energy of GO is somewhat higher than that of perovskite but lower than that of PANI and PESOT:PSS since all of these materials are in charge of transporting the holes. The LUMO increases from perovskite to PANI in a similar manner. By using ultraviolet photoelectron spectroscopy, the energy alignment of three ETLs, including pure SnO_2_, SnO_2_/PCBM, and SnO_2_/PCBA, was investigated (UPS) [62]. The secondary electrons shifted to lower binding energies when the two distinct passivation layers were integrated into SnO_2_. The operating function of the ETL was improved from that attributed to the base of the surface dipole by passivating these two layers. As a result, the surface dipole influences the electrical field in the electron transport layer (ETL), increasing electron collection at SnO_2_ and reducing damage from charge reconsolidation. The HOMO orientation is better maintained with the perovskite thanks to the passivation of PCBM and PCBA in SnO_2_, which may be helpful for charge transportation in an ETL/perovskite interface and increase efficiency [62]. Two ETLs, PCBM and 2FBT2FPDI, had their energy profiles analyzed [34]. The LUMO of 2FBT2FPDI is close to the conduction band of MAPbI_3_ (- (The LUMO of 2FBT2FPDI is close to that of PCBM (−3.90). As a result, a tiny energy gap was discovered at the 2FBT2FPDI/MAPbI_3_ interface, meaning that the MAPbI_3_ electron transit ratio is superior to the PCBM one.

**Figure 7:**
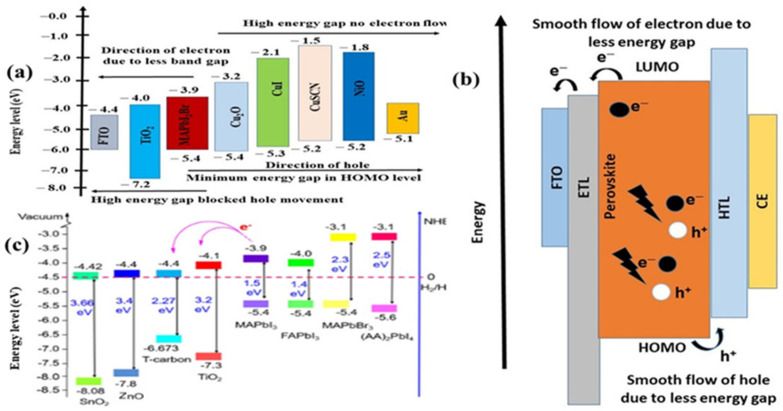
Energy level profile of perovskite solar cells. (**a**) Energy profile of the widely used materials of PSC with variable HTL. It shows the order of energy required to move electrons in an ETL and holes in HTL sides from the active layer. (**b**) Possible position of all the constituents of PSC with HOMO and LUMO. (**c**) the comparative energy level diagram of widely used ETLs (SnO2, ZnO, and TiO_2_) with an allotrope of Carbon (T-carbon) and different active materials (MAPbI_3_, FAPbI_3_, MAPbBr_3_, and (AA)_2_Pb I_3_) used in PSC. This diagram provides a clear picture of T-carbon, which has the same energy as most TiO2 and is considered a promising electron transporter [18].

When plasmonic nanoparticles (NPs) are introduced into a perovskite, hot electron transfer (HET) is injected by light in the direction of the ETL [116,119]. For instance, Au/SnO_2_ core-shell NPs contribute to improving the charge transfer from perovskite to ETL [7]. Near electromagnetic field (NEF)-encouraged charge transfer inside ETL is responsible for improving the solar cell’s performance. Au NPs produce HETs, which are in charge of breaking down the Schottky barrier between plasmonic NPs and ETL in order to release electrons into the latter and decrease the trap state, allowing plasmonic NPs to retain their equilibrium at the fermi energy level [7]. CsPbI_2.25_Br_0.75_ perovskite and CsAc@TiO_2_ NPs were used as the ETL in an HTL-free solar cell, and the energy profiles of the conduction band (CB) and valence band (VB) were analyzed [4]. The conduction bands of perovskite (-3.59 eV) and CsAc@TiO_2_ (-4.05) are well matched, meaning there is less of a gap between them. In order to increase efficiency and reduce damage from electron transmission from perovskite to TiO_2_ NPs, energy levels must be appropriately matched. Qiang et al. [30] utilized SnO_2_ as the ETL for CH_3_NH_3_PbI_3_-based HTL-free carbon PSC and discovered the perovskite’s CB and VB as −3.9 eV and −5.4 eV, respectively. Due to the perovskite layer’s ability to transition between an ETL or HTL state, this band gap gives the perovskite an ambipolar characteristic useful when building flexible solar cells. Nonetheless, SnO_2_’s CB and VB are more compatible than perovskites so they can transmit electrons more effectively. The LUMO of perovskite needs to have a large energy gap with the LUMO of HTL in order to block the transfer of electrons toward the hole sides. The energy gap between the LUMO of perovskite and ETL must be as small as possible [81]. In contrast, the HOMO energy gap between perovskite and ETL needs to be larger to stop transport holes from moving in that direction, whereas the HOMO energy gap between perovskite and HTL needs to be small to allow for proper hole movement in that direction. This type of alignment offers a better energy gap, which helps to increase efficiency and Voc [17,80,102]. The energy alignment for components of PSC with modification of ETL, HTL, Perovskite, position of HOMO and LUMO in PSC architecture are illustrated in Figure 7.

Gao et al. [70] fabricated carbon-based PSC using four-hole bridging materials such as carbon nanotube (CN), acetylene black (AB), nanocarbon (NC), and graphene (GN). The solar cell can be found on the ETL or HTL sides. Due to the low energy difference between perovskite and TiO_2_, electrons can quickly jump from the perovskite to the TiO_2_ CB. However, because of the wide energy gap, perovskite hole VB cannot jump to TiO_2_. Due to the perovskite’s VB and the bridging layers’ lower energy difference, holes in the perovskite shift toward the bridging layer or HTL. Perovskite’s VB is −5.32 eV, while the energy levels of bridging materials CN, AB, NC, and GN are in that order [70]. Solar cells cannot produce high Voc and Jsc if the electrons and holes migrate to unexpected sides. When unfavorable energy in the HTL to CE or ETL to FTO creates interfacial recombination, the significant energy differential, either in the ETL side or HTL side with regard to perovskite, causes bulk recombination. Perovskite/ETL or perovskite/HTL may also undergo interfacial recombination. As a result, this recombination can be prevented by adding polymer, carbon, and nanomaterials to the layer or by passivating it with a solvent.

## 7. Current Trends and Commercialization of PSCs

The efficiency of PSC (inorganic/organic PSC) has increased over the past ten years from 3.5% to 25.7%, having a similar efficiency to silicon solar cells that are commercially accessible [142]. Using new materials (FcTc_2_ and MS as perovskite passivates) in nano- and microscale, solution form, or solid form with the aid of various coating processes such as spin coating, doctor blade, screen printing, and many more is the best strategy to enrich efficacy. The performance of the PSC has been improved by materials such as 2FBT2FPDI, PCBM, SnO_2_-KCl, C_60_, and other composites and doping utilized in ETL [52]. Moreover, the materials used in HTL, such as Cu_2_O, CuI, CuSCN, spiro-OMeTAD, etc., have been praised for their high stability, excellent carrier mobility, and affordability [52]. Due to their strong interaction with Pb (Pb-O) and reduced bulk recombination of electrons and holes, light harvesting passivation layers such as FcTc_2_ and MS may offer the potential for the development of PSC in the future. Using ferrocene or metal units, including an electron-rich and electron-delocalized core atom, can enhance interfacial recombination. These materials can also have advantageous qualities such as stability at high humidity and the capacity to maintain most of their effectiveness when exposed to light for an extended period (A.M. 1.5 or 100 mWcm^−2^) [142]. The carbonaceous compounds are unreliable as efficiency enhancers; however, they offer reasonable stability, are inexpensive, and bring hydrophobicity in humid environments during rain and overcast days [89]. MWCNTs, EACQDs, biometric carbons, and their doping with NiO, CuS, GS, and CND are a few of the carbon and non-carbon-based CEs that may have a possibility for the commercial scale fabrication of PSC; however, Ag and Au are considered the most efficient CEs even though these have a high possibility for the commercial production of PSC [98]. The deposition of novel metal-based CE is challenging because it requires high thermal evaporation methods, thereby making it a difficult and costly method for the commercial-scale production of PSC. Transfer printing can be considered the better option for thermal evaporation due to its speed, strength, and usefulness for coating thin layers on other materials such as transistors and diodes [145]. The stability and efficacy of the solar cell using the transfer printing method are considered superior to thermal evaporation and other coating methods [145]. N-i-p-based perovskite solar cells have been prepared and deposited in a solvent (a mixture of dimethylformamide (DMF) and dimethyl sulfoxide (DMSO)), then injected with an anti-solvent (ethyl acetate) over 15 min. After that, the cell was dried for 20 min, followed by annealing [146]. This cell has incorporated poly[(9,9-dioctylfluorenyl-2,7-diyl)-co-(4,40-(N-(p-butylphenyl))diphenylamine)] (TFB) as a HTL. The cells treated with solvent and anti-solvent and kept dry before being taken for annealing were compared without keeping them dry. The efficiency of the cell in keeping dry has shifted from 10% to 17%. Therefore, this may be one of the novel methods to enhance solar performance, and it may have the possibility of commercial PSC in the future [146]. Furthermore, another novel approach is to produce lead-free PSC. This approach incorporated fully inorganic halide perovskite, i.e., cesium tin triiodide (B-γ CsSnI_3_), as an active layer. This perovskite has superior optoelectronic characteristics such as light absorption coefficient, electron-hole mobility, stability, and less excitation binding energy than lead halide perovskite. The best efficiency of this B-γ CsSnI_3_ to date is 10.1%, and it retained 94% initial stability over 60 days under 1 sun illumination at 70 °C temperature [147]. However, incorporating organic and inorganic materials, doping, composites of the materials, solvent, anti-solvent treatment, printing method, device drying, and annealing may raise efficacy and stability. These could lead PSC toward commercialization.

## 8. Conclusions

This study has included the different generations of solar cells, and their comparative advantages and disadvantages for the best efficiency are briefly discussed and illustrated in flow diagram 1. Additionally, among solar energy, the most attractive energy generation is perovskite solar and its performance with modification (ETL, HTL, perovskite, and CE) by variable novel materials and commonly used materials in comparison to solar parameters. Moreover, the hindrances that restricted the higher performance and stability of PSC have been included in the solution. For enhanced PSC fabrication, nanomaterials such as carbonaceous and non-carbonaceous plasmonic NPs and their performance have been included with their optical and electrical properties (Table 4), size, morphology, crystallinity, and energy alignment (Figure 7). Innovative methods such as surface passivation by precursor solutions, doping, and composites are quite successful in terms of stability and efficiency. The modification of such materials as ETL (Table 1), HTL, perovskite, and CE is critically discussed based on the other literature available. Furthermore, the effect of all layers in PSC on solar performance is also discussed and illustrated in tabular form (Table 3). Other factors, such as the effect of sintering temperature, types and speeds of coating, and the effect of the thickness of the layers, have also been reported, and the variation of these factors based on PSCs has been compared. The J-V characterization of the best materials—novel, efficient materials with a variable ratio of doping, passivation, and applied temperature—is discussed and illustrated by associating all the popular layers (Table 5). Finally, some current trends and commercialization possibilities of PSC have been briefly discussed. It was found that a few materials have proven themselves compatible materials in order to enhance the solar cell’s performance, but a few have been modified by doping, composites, and applied favorable conditions to upsurge the efficiency.

## Figures and Tables

**Table 1:** The variable materials used as ETL for PSC with different ratios of solvent, doping, thickness of cell, and applied temperature in terms of solar parameters.

ETL Materials	Thickness	Sintering Temperature	Types of Solar Cells	Voc (mV)	Jsc (mA cm^−2^)	FF%	η%	Ref.
SnO_2_: ethanol (1:0)	400 nm	200 °C for 1 h	Planar	860	20.84	38	6.78	[49]
SnO_2_: ethanol (1:1)				890	21.00	41	7.67	
SnO_2_: ethanol (1:2)				920	21.33	39	7.59	
SnO_2_: ethanol (1:3)				930	22.50	40	8.32	
SnO_2_: ethanol (1:4)				870	21.79	38	7.18	
SnO_2_: ethanol (1:5)				680	19.95	32	4.35	
m-TiO_2_: ethanol (1:6)	60 nm	20 °C @ 10 min	Planar	690	16.39	53.06	6.67	[33]
		40 °C @ 10 min		893	20.11	49.81	9.94	
		60 °C @ 10 min		964	18.89	54.94	11.12	
		80 °C @ 10 min		817	18.40	57.83	9.66	
		100 °C @ 10 min		851	14.40	53.55	7.29	
Na_2_S + TiO_2_ (0.5% solution)	-	450 °C @ 1 h	Planar	1210	22.63	75.10	19.85	[47]
Na_2_S + TiO_2_ (1% solution)				1220	23.85	75.35	21.25	
Na_2_S + TiO_2_ (2% solution)				1210	23.90	72.53	19.90	
Na_2_S + TiO_2_ (3% solution)				1190	23.58	73.25	18.80	
Pristine TiO_2_				1190	22.63	71.95	18.24	
TiO_2_ doped with 1% NaCl				1200	23.44	75.05	19.08	
SnO_2_ (Reverse scan)	40 nm	Dry the film @ 50 °C for 5 min	Planar	1080	22.58	75.79	18.52	[50]
SnO_2_ (Forward scan)				1010	22.58	72.48	16.53	
(CH_3_)_2_Sn(COOH)_2_ (Reverse Scan)				1160	23.89	74.23	20.57	
(CH_3_)_2_Sn(COOH)_2_ (forward Scan)				1140	23.84	73.04	19.85	
(CH_3_)_2_Sn(COOH)_2_ with KCl (Reverse Scan)	300 nm	Annealed @ 120 °C for 15 min		1180	23.93	78.23	22.09	
(CH_3_)_2_Sn(COOH)_2_ with KCl (forward Scan)				1180	24.03	78.33	22.21	
SnO_2_ (Reverse scan)	-	150 °C for 30 min in air	Planar	1077	24.0	77.9	20.2	[51]
SnO_2_ (Forward scan)				1017	24.0	75.4	18.4	
SnO_2_-KCl (Reverse scan)				1137	24.2	80.7	22.2	
SnO_2_-KCl (Forward scan)				1097	24.2	79.9	21.2	
Pristin PCBM	No passivation	-	Inverted	1080	22.7	70.0	17.3	[52]
2FBT2FPDI	7 nm			1100	23.5	72.5	18.8	
2FBT2FPDI	13 nm			1100	23.9	77.2	20.3	
2FBT2FPDI	21 nm			1110	22.8	72.0	18.2	
Pristine 2FBT2FPDI	-			1100	22.5	67.8	16.8	
PC_61_ BM	85 nm	80 °C each for 15 min	Planar	1033	22.05	70	15.43	[53]
BPy-C_60_				980	22.31	67	14.59	
BpAn-C_60_				989	22.05	59	12.92	
BAn-C_60_				851	16.93	24	3.42	
ZnO (forward scan)	300 nm	150 °C for 30 min in ambient air	Planar	971	20.12	66.4	12.93	[54]
ZnO (reverse scan)				996	20.63	67.3	13.82	
MLG/ZnO (forward)				1114	22.74	77.1	19.54	
MLG/ZnO (backward)				1120	22.71	77.9	19.81	
MLG/ZnO with passivation-(forward)				1149	23.39	77.5	20.82	
MLG/ZnO with passivation–(backward)				1150	23.42	78.1	21.03	

**Table 2:** Passivation of the perovskite layer with different materials in ETL and HTL side for testing stability under humidity and temperature in tested time intervals.

Passivated Material	Percentage Initial Stability of Efficiency	Thermal Ageing (Humidity)	Time (Hours)	Percentage Final Stability of Efficiency	Ref.
Without BETAB	90%	@ 60 °C	200	85%	[67]
BETAB	90% in air		500	90%	
Without F-PEAI	93.3%	@ 60 °C	720	70%	[68]
F-PEAI	98.9%			89.9%	
IDTT-ThCz	100%	@85 °C	500	95%	[69]
PEAI	Stable	@85 °C	Above 500	Stability declined after a few hours	[70]
CF_3_PEAI	100%	70–80% humidity	528	98%	[71]
PSK/CuSCN/C	100%	75–85%	240	98%	[6]
PSK/C-CuSCN	100%			91%	

**Table 3:** The variable carbon materials used in CE with common ETL and HTL (carbon and non-carbonaceous, doping, and composite structures) in terms of comparative solar parameters.

Carbon Material (CE)	ETLs	HTLs	Active Area (cm^2^)	V_oc_ (mV)	J_sc_ (mA cm^−2^)	FF (%)	ɳ (%)	Ref.
CNC	SnO_2_	CNC	1	936	15.34	33.28	4.78	[58]
MWCNT		MWCNT		1009	22.01	50.44	11.20	
Graphene		Graphene		996	21.93	41.96	9.17	
Pure carbon(C)	c-TiO_2,_ m-TiO_2_	C	0.16	1018	17.59	57.5	10.29	[93]
NiO:Carbon (NiO-C) (1:10)		NiO-C		1021	19.95	60	11.80	
NiO:Carbon (1:20)		NiO-C		1035	20.93	61.12	13.26	
NiO:Carbon (1:30)		NiO-C		1030	20.15	60.7	12.60	
Carbon (C)	c-TiO_2,_ m-TiO_2_	C	0.14	930	16.14	62.69	9.36	[2]
C-0.5% CuS		(C-CuS)		920	16.62	63.92	10.14	
C-1% CuS		(C-CuS)		980	18.26	63.23	11.28	
C-2% CuS		(C-CuS)		880	16.67	57.87	8.49	
Coal-C coating	c-TiO_2,_ m-TiO_2_	Coal-C	1.0	890	22.29	44	8.72	[40]
Carbon Black (CB)		CB	0.3	840	21.39	60	10.87	
Standard carbon electrode (std)	c-TiO_2,_ m-TiO_2_, m-ZrO_2_		0.49	830	3.35	46.68	7.73	[94]
Std + carbon ink (SCI)		SCI		840	3.53	43.44	7.70	
Std + carbon ink + Cu grid (SCI-Cu)		(SCI-Cu)		840	3.65	59.18	11.05	
Std + carbon ink + Al grid (SCI-Ag)		(SCI-Ag)		850	3.49	56.77	9.97	
CS-B	TiO_2_	CS-B	0.15	690	18.70	49	6.36	[16]
PA-B		PA-B		740	19.35	54	7.85	
PS-B		PS-B		81	21.06	61	10.30	
BC-B		BC-B		85	23.15	65	12.82	
Pristine	PCBM	NiO_x_	0.06	1040	18.24	76.36	15.11	[95]
3% CND				1040	19.38	73.94	15.86	
5% CND				1060	20.13	77.34	16.94	
10% CND				1010	18.68	74.55	15.32	
Urea				1050	21.57	74.98	17.76	
5% CND/Urea				1070	22.74	76.92	19.50	
CN/carbon	TiO_2_	CN	0.06	940	23.51	68	15.09	[96]
AB/carbon		AB		910	22.99	64	13.47	
NC/carbon		NC		890	22.88	61	12.37	
GN/carbon		GN		850	22.11	61	11.50	
EACQDs (pristine)	c-TiO_2_, m-TiO_2_, ZrO_2_	Carbon quantum dots (CQDs)	0.06	1005	21.65	55.86	12.15	[97]
EACQDs (0.005 mg/mL)				1016	22.49	60.91	13.92	
EACQDs (0.01 mg/mL)				1021	22.72	65.29	15.14	
EACQDs (0.02 mg/mL)				1002	22.35	61.86	13.87	
EACQDs (0.05 mg/mL)				9880	21.10	59.38	12.38	
CB	SnO_2_	Spiro-OMeTAD	1.30	1040	21.11	64	14.05	[98]
GS				840	21.52	55	9.94	
G				1050	22.78	78	18.65	
Carbon black (CB)	SnO_2_	w/N-CQDs	0.09	1622	7.87	80.1	10.71	[14]
		w/o N-CQDs		1588	7.64	84.1	10.20	

**Table 4:** The comparative values of R_s_ and R_ce_ of the materials used in CE in terms of solar parameters.

ETL/Perovskite/CE Interface	(R_s_) Ω	(R_rce_) Ω	Voc (mV)	Jsc (mAcm^−2^)	FF (%)	η (%)	Ref.
Cf	40.71	306.90	780	21.32	45	7.47	[12]
CNTs	32.84	203.90	800	22.98	49	8.93	
CNTs/Cf	27.84	102.30	770	23.60	65	11.80	
O-MWCNTs (0.0 mg/mL)	26.2	577	970	21.89	37.24	7.96	[110]
O-MWCNTs (0.5 mg/mL)	25.8	833	996	21.96	41.09	8.99	
O-MWCNTs (1.0 mg/mL)	28.8	93	841	19.42	32.58	5.28	
VC	11.1	2.8	940	20.87	63.77	12.55	[124]
MC	10.7	1.6	930	20.53	61.28	11.76	
SP	12.8	15.0	940	20.22	55.07	10.48	
Au@Ag@SiO_2_ (0%)	23.49	131.8	1033	20.63	70	15.41	[116]
Au@Ag@SiO_2_ (0.5%)	23.73	169.0	1046	21.02	71	16.12	
Au@Ag@SiO_2_ (1.0%)	27.68	184.1	1044	22.10	72	17.38	
Au@Ag@SiO_2_ (1.5%)	28.04	240.5	1048	21.15	71	16.15	

**Table 5:** Comparative study of four constituents of PSC such as ETL, HTL, perovskite and CE, in terms of solar cell parameters.

Substrate	ETL	Perovskite	HTL	CE	Voc (mV)	Jsc (mAcm^−2^)	FF (%)	ɳ (%)	Ref.
FTO	TiO_2_	MAPbI_2_Br	NiO	Au	720	23.28	50.85	8.53	[139]
			Cu_2_O		1292	23.30	83.65	25.21	
			CuI		1138	23.33	72	21.80	
			CuSCN		950	23.30	59.91	13.35	
FTO	TiO_2_ treated with TiCl_4_ (no annealing)	CH_3_NH_3_PbI_3_	Spiro-OMeTAD	Ag	947	19.16	49.87	9.09	[140]
	Annealed @ 150°C				1016	21.57	64.65	14.16	
	Annealed @ 300°C				975	19.99	53.45	10.42	
	Annealed @ 450°C				990	20.49	58.91	11.98	
FTO	TiO_2,_ PANI was incorporated at TiO_2_/MAPbI_3_ Interface	MAPbI_3_	Spiro-OMeTAD	Au	960	17.95	62.1	10.7	[141]
		PANI/MAPbI_3_ (65 nm)			990	23.71	64.2	15.1	
		PANI/MAPbI_3_ (100 nm)			980	20.10	64.1	12.7	
		PANI/MAPbI_3_ (135 nm)			980	13.40	63.2	11.0	
		PANI/MAPbI_3_ (170 nm)			980	16.30	64.4	10.3	
ITO	C_60_	Cs_0.05_ (FA_0.98_MA_0.02_)_0.95_Pb(I_0.98_Br_0.02_)_3_ (control)	Poly (triaryl- amine) (PTAA)	Ag	1130	25.25	80.45	23.0	[142]
		Cs_0.05_ (FA_0.98_MA_0.02_)_0.95_Pb(I_0.98_Br_0.02_)_3_/FcTc_2_ (1.0 mg/mL)			1184	25.68	82.32	25.0	
FTO	TiO_2_	FA_0.85_MA_0.15_PbI_3_ (control)	Spiro-OMeTAD	Au	1090	24.02	71.91	19.55	[72]
		FA_0.85_MA_0.15_PbI_3_/TPFPB			1120	24.69	78.22	21.60	
ITO	SnO_2_	FAPbI_3_(control)	Spiro-OMeTAD	Au	1136	26.27	78.49	23.42	[143]
		FAPbI_3_/MS			1164	26.31	82.82	25.38	
ITO	SnO_2_	CsPbBrI_2_/BMIMBF_4_ (0 mg/mL)	Carbon	Carbon	1140	14.33	69	11.37	[137]
		CsPbBrI_2_/BMIMBF_4_ (0.25 mg/mL)			1270	14.21	72	13.09	
		CsPbBrI_2_/BMIMBF_4_ (0.50 mg/mL)			1270	14.68	75	14.03	
		CsPbBrI_2_/BMIMBF_4_ (1.0 mg/mL)			1270	14.30	71	12.88	
		CsPbBrI_2_/BMIMBF_4_ (2.5 mg/mL)			1260	13.88	66	11.49	
		CsPbBrI_2_/BMIMBF_4_ (5 mg/mL)			1250	13.78	58	10.02	
